# Optimized MaxEnt Models Predict Species-Specific Habitat Suitability Shifts for Three Dryland Fabaceae Species in Xinjiang

**DOI:** 10.3390/plants15152254

**Published:** 2026-07-23

**Authors:** Jiaqi Chen, Qilong Tian, Qianqian Ma, Rongbin Li, Qingcuo Ren

**Affiliations:** 1 Key Laboratory of Oasis Ecology, Ministry of Education, College of Ecology and Environment, Xinjiang University, Urumqi 830017, China; 107552405016@stu.xju.edu.cn (J.C.); lirongbin@stu.xju.edu.cn (R.L.); 20241701203@stu.xju.edu.cn (Q.R.); 2 State Key Laboratory of Soil and Water Conservation and Desertification Control, The Research Center of Soil and Water Conservation and Ecological Environment, Chinese Academy of Sciences and Ministry of Education, Xianyang 712100, China; 3 College of Soil and Water Conservation Science and Engineering, Northwest A&F University, Xianyang 712100, China

**Keywords:** MaxEnt, dryland legumes, habitat suitability, niche overlap, climate change

## Abstract

Climate warming is altering plant range limits in drylands, where water limitation, soil salinity, and thermal extremes jointly constrain species niches. Yet it remains unclear whether dryland legumes with contrasting life forms respond differently to future climates, a problem that remains poorly resolved. We used ENMeval-optimized MaxEnt models to estimate the current and future potential distributions of three ecologically important legumes in arid Xinjiang: *Alhagi camelorum*, a perennial semi-shrub; *Glycyrrhiza inflata*, a perennial herb; and *Trigonella arcuata*, an annual herb. Models were evaluated under four Shared Socioeconomic Pathways for 2061–2080 and 2081–2100 and showed high discriminatory performance with test AUC values of 0.910–0.947. Under the current climate, *G. inflata* formed a concentrated core in the Tarim Basin oases, *T. arcuata* showed a fragmented northern-biased distribution, and *A. camelorum* was widely dispersed across both northern and southern Xinjiang. The strongest predictors differed among species: minimum temperature of the coldest month for *G. inflata* (45.9% contribution), precipitation seasonality for *T. arcuata* 76.8%, and NDVI for *A. camelorum* 34.0%. Bootstrap-based niche analyses further quantified these differences: *A. camelorum* and *G. inflata* had similar niche breadths, Levins’ B2 = 0.329 and 0.315, respectively, whereas *T. arcuata* had the narrowest niche breadth, B2 = 0.225; 95% CI: 0.162–0.241. Pairwise niche overlap was generally low, particularly between *G. inflata* and *T. arcuata* (Schoener’s D = 0.019; Hellinger’s I = 0.038). Projections based on BCC-CSM2-MR indicated species-specific and scenario-dependent suitability shifts across both future periods. *G. inflata* showed the largest projected net gains across periods and scenarios; *A. camelorum* showed moderate but consistently positive gains, whereas *T. arcuata* showed weaker and more uncertain responses, including significant net losses under SSP1-2.6. These asymmetric trajectories suggest species-specific responses among the three legumes and provide hypotheses for how life-form-related ecological strategies may shape dryland habitat shifts. Given this limited replication, these trajectories should be treated as species-specific hypotheses rather than evidence for life-form effects. Future SDM assessments should broaden taxonomic sampling and incorporate key functional traits to improve forecasts of dryland community reassembly and conservation planning.

## 1. Introduction

Climate change is increasingly threatening biodiversity and ecosystem services worldwide, with especially severe effects in arid and semi-arid regions, where ecosystems are fragile and highly sensitive to environmental perturbation [1,2]. The climate-driven redistribution of plant species has therefore become a central process linking biogeography, regional ecological security, and the sustainability of pastoral livelihoods [3]. Understanding how species distributions respond to climate change is now a key frontier in biogeography and conservation biology, with direct relevance to predictive theory and adaptive management [4]. Global assessments identify climate-driven range shifts as an important constraint on progress towards Sustainable Development Goals 13, Climate Action, and 15, Life on Land [5].

Species distribution models, particularly Maximum Entropy models, are widely used to project potential habitats under future climate scenarios [6,7,8,9]. Early applications relied largely on correlations between species occurrences and climatic variables; subsequent studies incorporated topographic, edaphic, and remotely sensed predictors to better capture environmental heterogeneity relevant to species distributions [10]. Correlative SDMs, however, remain sensitive to occurrence sampling bias, background selection, accessible-area definition, spatial autocorrelation, and extrapolation into non-analog climates. Systematic model tuning, by optimizing regularization multipliers and feature-class combinations with information criteria such as AICc, can reduce overfitting and improve transferability [11,12]. These developments have reinforced the view that climatic extremes, edaphic constraints, and topographic heterogeneity jointly structure species distributions. Their relative importance, however, may vary sharply among species, especially in drought-prone ecosystems where water limitation and salinity impose strong physiological constraints.

Recent research has focused increasingly on how plants that live in deserts and dry areas react to changes in the climate. Models of species distribution for ephemeral plants in arid and semi-arid regions around the world show that temperature, precipitation, and soil-related factors can have a big effect on predicted range shifts [13]. In northwestern China, climate factors also shape desert plant biomass and species richness in northern Xinjiang [14]. MaxEnt-based studies have further evaluated future habitat dynamics for dominant desert species in Xinjiang and Central Asia, including *Haloxylon ammodendron*, *Ephedra species*, and other desert taxa [15,16,17]. For legumes, recent work on *Glycyrrhiza inflata* indicates that climate change and land-use pressure may substantially affect suitable habitats, underscoring the ecological and conservation importance of dryland Fabaceae [18]. Yet most studies in Xinjiang and Central Asian drylands have focused on single species, medicinal plants, or dominant desert taxa. Evidence remains limited on whether taxonomically related dryland legumes occupying the same regional environmental space show comparable or contrasting associations with climatic, edaphic, and topographic conditions [13,14,15,16,17,18,19]. This gap is important because species within the same family may differ in persistence, regeneration, resource acquisition, and tolerance of environmental stress, even when they occur within broadly similar dryland ecosystems [20,21]. A species-level comparative framework can therefore identify whether projected suitability shifts correspond to distinct environmental filters and niche characteristics without attributing such differences causally to life form or to any single functional trait.

The Xinjiang Uygur Autonomous Region, located in northwestern China and in the heart of Central Asia, spans 34–49° N and 73–96° E and covers approximately 1.66 million km^2^. It is characterized by a “three mountains and two basins” topography and a temperate continental arid climate [22]. The region provides an informative natural laboratory for examining legume distribution dynamics for three main reasons. It is highly climate-sensitive, with warming rates exceeding the global mean [23]; it contains strong environmental gradients, including extreme drought, soil salinization, and elevation-dependent temperature variation; and it is subject to substantial anthropogenic pressure from agricultural expansion and large-scale ecological engineering [24]. Fabaceae play important ecological roles in drylands through symbiotic nitrogen fixation, which improves soil fertility and supports livestock production [25]. Xinjiang harbors multiple legume species with contrasting ecological strategies, including *Alhagi camelorum*, a perennial semi-shrub; *Glycyrrhiza inflata*, a perennial herb with persistent belowground structures; and *Trigonella arcuata*, an annual herb. These species provide a tractable system for comparing species-specific habitat-suitability responses within one plant family under shared regional environmental conditions.

These considerations motivate a comparative species-level assessment that links environmental filtering, niche structure, and projected climate-driven suitability change while avoiding causal inference about life-form effects. We therefore combined systematically optimized MaxEnt models with a comparative species-level framework to ask: (1) whether current habitat-suitability patterns of the three legumes are associated with different dominant environmental filters; (2) whether the species differ in niche breadth and environmental overlap within the same regional dryland system; and (3) whether projected future habitat-suitability shifts differ among species under alternative climate scenarios. We expected the three species to show distinct dominant environmental correlates, niche characteristics, and scenario-dependent suitability changes because they occupy different portions of the regional environmental space. These comparisons were designed to evaluate species-specific patterns, not to test causal effects of life form, because each life-form category was represented by only one species.

## 2. Materials and Methods

### 2.1. Study Area

**Figure 1:**
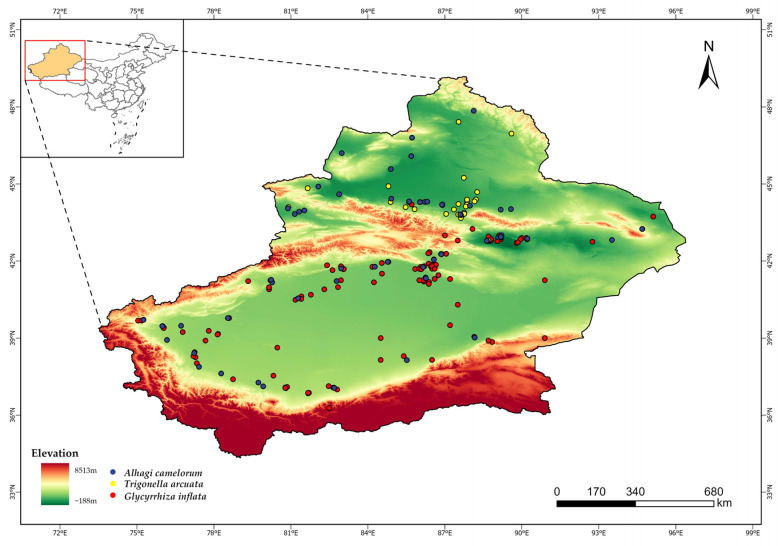
Spatial distribution of occurrence records of the three legume species in Xinjiang. The administrative boundaries of the Xinjiang Uygur Autonomous Region were derived from the standard map downloaded from the Standard Map Service of the Ministry of Natural Resources of the People’s Republic of China (review number GS(2024)0650), and the base-map boundaries were not modified.

The Xinjiang Uygur Autonomous Region is located in northwestern China (34°25′–49°10′ N, 73°40′–96°23′ E), spans approximately 1.66 million km^2^, and encompasses pronounced environmental heterogeneity [26]. The three focal legume species—*Alhagi camelorum*, *Trigonella arcuata*, and *Glycyrrhiza inflata*—occur mainly in the region’s arid and semi-arid zones, particularly in the Tarim Basin, the Junggar Basin, and the foothills of the Tianshan Mountains (Figure 1). Xinjiang has a temperate arid continental climate, with a mean annual temperature of 4–12 °C, varying strongly with elevation, and annual precipitation of 50–250 mm, which generally declines from mountain areas towards basin interiors. Topography grades from the high-altitude Tianshan, Kunlun, and Altai Mountains to the low-lying Taklamakan and Gurbantunggut Deserts. This relief generates strong temperature-moisture gradients, marked soil-salinity variation, and diverse ecological settings, ranging from montane meadows to hyper-arid desert steppes. Together, these environmental gradients provide a suitable framework for evaluating how the potential habitats of the three legumes may shift under future climate scenarios. Because background extent can influence MaxEnt calibration and evaluation, Xinjiang was used as the initial modeling domain to represent the regional species pool. However, we recognize that this administrative boundary may exceed the accessible area of some species, particularly those with restricted northern or southern distributions.

The three focal legumes differ markedly in life history, belowground resource-acquisition strategy, and regeneration mode, all of which are relevant to their responses to climate and habitat conditions. *Alhagi camelorum* is a long-lived perennial semi-shrub. Ecological studies of camelthorn populations in Xinjiang, particularly those identified as *A. camelorum*, have described a deep-rooted phreatophytic strategy, strong drought adaptation, and persistence in desert–oasis transition habitats [27]. *Glycyrrhiza inflata* is a perennial herb with well-developed underground rhizomes and can reproduce both sexually by seeds and vegetatively through belowground rhizomes; these traits may facilitate persistence under seasonal stress and in fragmented oasis habitats [28]. In contrast, *Trigonella arcuata* is an annual or ephemeral desert legume that depends on seasonal establishment and forms symbiotic associations with rhizobia adapted to desert environments [29]. These differences in life history, rooting strategy, regeneration mode, and stress-related adaptation provide the functional basis for comparing species-specific responses to environmental gradients and future climate change within dryland Fabaceae. A summary of the key functional traits of the three focal species is provided in Table S5.

### 2.2. Study Framework

**Figure 2:**
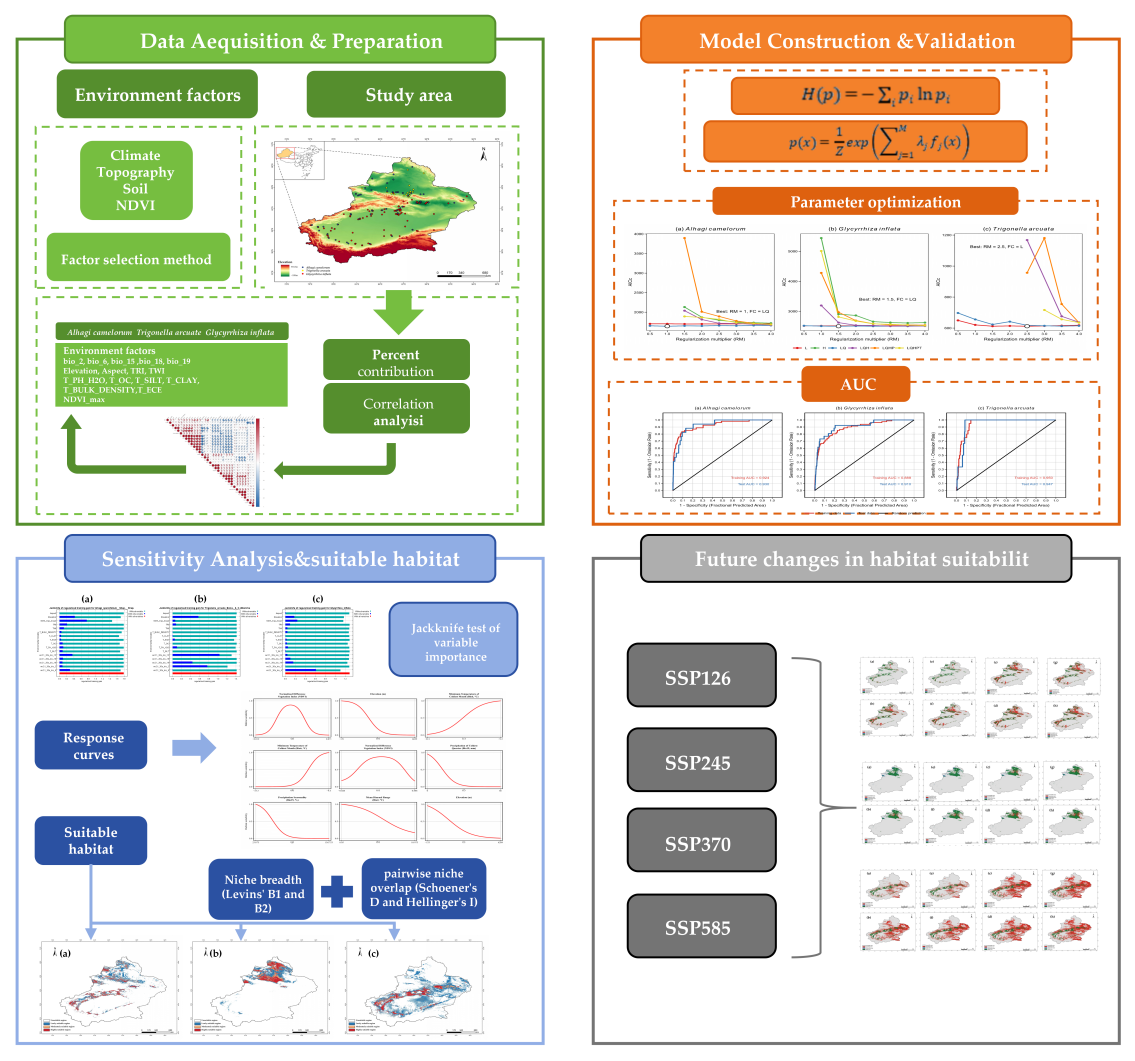
Workflow of the research framework, comprising four sequential stages: (1) occurrence-data collection, quality control, and spatial thinning; (2) environmental-variable screening and preparation; (3) MaxEnt model optimization, validation, and niche analysis; and (4) current and future habitat-suitability projections, habitat-change classification, and uncertainty assessment under different SSP scenarios.

The study followed a four-step workflow (Figure 2). First, occurrence records for the three species were compiled, quality controlled, spatially thinned, and matched with 33 candidate environmental predictors, of which 16 were retained after correlation and VIF screening. Second, species-specific MaxEnt models were optimized using ENMeval v2.0.5.2 and evaluated using non-spatial and spatial validation procedures. Third, variable importance, response curves, current suitability patterns, niche breadth, and niche overlap were quantified. Finally, habitat suitability was projected for 2061–2080 and 2081–2100 under four SSP scenarios, and changes were classified as stable unsuitable, expansion, contraction, or stable suitable habitat using species-specific MaxTSS thresholds. Sensitivity, bootstrap, and randomization analyses were used to evaluate uncertainty in model performance, niche metrics, and projected habitat change.

### 2.3. Data Processing

#### 2.3.1. Occurrence Data Collection

Georeferenced occurrence records for *Alhagi camelorum*, *Trigonella arcuata*, and *Glycyrrhiza inflata* were compiled from the Chinese Virtual Herbarium (CVH; http://www.cvh.ac.cn, accessed on 1 March 2026), the Global Biodiversity Information Facility (GBIF; https://www.gbif.org, accessed on 1 March 2026), and peer-reviewed literature documenting occurrences in Xinjiang. Before modeling, records underwent a standardized quality-control procedure [30]. Species names and synonyms were harmonized across sources, and duplicate records, including those shared among sources, were removed. Records were excluded if coordinates were missing or imprecise, locality descriptions were ambiguous, locations fell outside Xinjiang, or coordinates were clearly erroneous. The remaining records were visually inspected in ArcGIS Pro 3.0.2 against administrative boundaries, elevation layers, and documented regional distributions to identify residual outliers. This procedure retained 216 records for *A. camelorum*, 51 for *T. arcuata*, and 222 for *G. inflata*. To reduce spatial autocorrelation and local sampling clusters [31], occurrence records were spatially thinned using the “Spatially Rarefy Occurrence Data for SDMs” tool in ArcGIS Pro 3.0.2. We selected a 5 km thinning distance to balance bias reduction against retention of occurrence information. This distance exceeds the resolution of the 30 arc-second environmental layers, reduces pseudo-replication among neighboring records, and limits clustering around roads, settlements, and oasis areas, where herbarium and database records are often concentrated [32,33,34]. After thinning, 74 records of *A. camelorum*, 29 of *T. arcuata*, and 114 of *G. inflata* remained for model calibration.

Because spatial thinning alone cannot fully account for uneven collection effort, we assessed potential sampling bias using a sampling-effort null model. A sampling-effort surface was generated from pooled pre-thinning occurrence records obtained from CVH, GBIF, and the literature. Records were rasterized, smoothed using kernel-density estimation, resampled to the resolution of the environmental predictors, and standardized to values between 0 and 1. Then, for each species, 999 null datasets, each containing the same number of records as the final thinned dataset, were sampled from the sampling-effort surface. The observed mean sampling-effort value and mean nearest-neighbor distance were compared with their respective null distributions to determine whether occurrence records were more strongly associated with collection effort or more spatially clustered than expected under the regional sampling pattern. The sampling-effort surface and final thinned occurrence records are shown in Figure S2.

#### 2.3.2. Environmental Data Sources and Preprocessing Procedures

Current climatic variables (1970–2000) and future climate projections were obtained from WorldClim v2.1 at a 30 arcsec resolution (https://www.worldclim.org/data/worldclim21.html, accessed on 1 March 2026) [35]. Future bioclimatic variables were derived from downscaled CMIP6 projections for the BCC-CSM2-MR general circulation model under SSP1-2.6, SSP2-4.5, SSP3-7.0, and SSP5-8.5 for 2061–2080 and 2081–2100. These scenarios span a gradient from low-emission, sustainability-oriented development to fossil-fuel-powered development with very high emissions. Owing to the pronounced time lag in the response of ecosystems to climate change, the 2061–2100 period was selected to assess the long-term potential for habitat shifts and adaptability thresholds under cumulative climatic effects [36]. BCC-CSM2-MR reproduces broad temperature and precipitation patterns across Eurasia and northwestern China, although uncertainty remains for precipitation estimates and topographically complex areas [37,38]. Future projections were therefore interpreted as conditional estimates based on a single climate model, rather than as multi-model consensus forecasts. Nineteen bioclimatic variables were extracted from WorldClim. Topographic predictors, including elevation, slope, aspect, terrain ruggedness index (TRI), terrain roughness, and topographic wetness index (TWI), were derived from the SRTM digital elevation model. Soil variables were obtained from the Harmonized World Soil Database (HWSD v1.2; https://www.fao.org/soils-portal/data-hub/soil-maps-and-databases/harmonized-world-soil-database-v12/en/, accessed on 1 March 2026), including topsoil pH, soil organic carbon (SOC), sand, silt, clay, bulk density (BD), and electrical conductivity (ECE). Vegetation productivity was represented by mean annual maximum NDVI for 2000–2024. Monthly NDVI data were obtained from the MODIS MOD13A3 product [39]. For each year, annual maximum NDVI was calculated as the highest monthly value and subsequently averaged across 2000–2024.

Because NDVI data contemporaneous with the 1970–2000 climatic baseline was unavailable at a comparable resolution, the NDVI layer represented recent vegetation productivity rather than conditions strictly synchronous with the baseline climate. Current predictions were therefore interpreted as potential suitability conditional on historical climate normals and recent vegetation conditions. For future projections, only the bioclimatic variables were replaced with CMIP6 projections. Topographic variables were retained because they are effectively static at the spatial and temporal scales considered, whereas soil variables and NDVI were retained as present-day baseline constraints because scenario-consistent future layers were unavailable. Future predictions thus represent climate-driven changes in potential suitability conditional on present-day soil, terrain, and vegetation-productivity backgrounds. The influence of the temporally mismatched NDVI layer was assessed using an NDVI-exclusion sensitivity analysis (Section 2.4.2). We initially assembled 33 candidate predictors representing climatic, topographic, edaphic, and vegetation-productivity constraints in Xinjiang drylands (Table S1). These variables captured thermal extremes, precipitation regimes, topographic heterogeneity, soil salinity and texture, soil organic carbon, and vegetation productivity. To reduce multicollinearity and model complexity, predictors were screened in two steps. First, highly correlated predictor pairs were identified using Pearson correlation coefficients (|r| > 0.8), and the variable considered less ecologically relevant to dryland legume ecology was removed. Second, variables with variance inflation factors (VIFs) > 10 were iteratively excluded until all retained predictors had VIF ≤ 10. This procedure retained 16 non-collinear and ecologically interpretable predictors for comparative MaxEnt modeling (Table 1). Correlation matrices and VIF-screening results are provided in Figure S1 and Tables S2–S4. All layers were clipped to Xinjiang, resampled to a 30 arc-second resolution, and converted to ASCII format in ArcGIS Pro 3.0.2 before model calibration.

### 2.4. Methods

#### 2.4.1. Principle of the MaxEnt Model

This study used MaxEnt v3.4.4, based on the maximum-entropy principle, to predict the potential distribution of the three legume species. The core concept is to maximize information entropy subject to constraints that the expected values of environmental features, as estimated from species-occurrence samples, equal those under the model [6]. The maximum-entropy probability distribution can be expressed as (Equation (1)):

(1) $$H\left(p\right)=-\sum_{i}p_{i}\ln p_{i}$$

The resulting “most uniform” (least assumptive) probability distribution takes the form as follows (Equation (2)):

(2) $$p\left(x\right)=\frac{1}{Z}exp\left(\sum_{j=1}^{M}\lambda_{j}f_{j}\left(x\right)\right)$$

where *fj*(*x*) denotes the jth environmental feature function, *lj* is the corresponding Lagrange multiplier, and *Z* is the normalizing constant. This approach avoids making unwarranted inferences when data are limited and reduces overfitting risk. MaxEnt also integrates a jackknife procedure to estimate the relative importance of each predictor, enabling variable-importance ranking, and supports multiple feature classes (linear, quadratic, hinge, product, and threshold) for model optimization.

#### 2.4.2. Species Distribution Models

MaxEnt v3.4.4 was used to model the relative habitat suitability of *A. camelorum*, *T. arcuata*, and *G. inflata* using the 16 retained environmental predictors. Models were fitted with logistic output, yielding relative suitability values from 0 to 1. For the primary models, occurrence records were randomly split into training (75%) and test (25%) subsets [40], with a maximum of 1000 iterations. We generated 10,000 random background points within Xinjiang to characterize the environmental background of the regional modeling domain [41]. Final regularization multiplier (RM) and feature-class (FC) settings were selected separately for each species using ENMeval, as described in Section 2.4.3.

Three sensitivity analyses were conducted to assess the influence of key modeling assumptions. First, to evaluate collection bias, background points were sampled according to the standardized sampling-effort surface rather than randomly across Xinjiang. Bias-corrected and primary models were compared in terms of performance, MaxTSS threshold, and estimated current suitable area (Table S14). Second, because background extent may influence model calibration, a restricted-domain analysis was conducted for *T. arcuata*. A 100 km buffer around spatially thinned occurrence records, clipped to Xinjiang, was used as a pragmatic restricted-domain proxy rather than a definitive estimate of the accessible area (M). Models calibrated within this restricted domain were compared with Xinjiang-wide models (Table S9). Third, to assess the effect of the temporal mismatch between NDVI and baseline climate data, NDVI was excluded, and models were re-tuned using the remaining 15 predictors. Full and NDVI-excluded models were compared in terms of optimal parameterization, AUC, MaxTSS, and current binary suitable area (Table S10).

Model discrimination was assessed using test AUC and the true skill statistic (TSS). AUC quantified discrimination between occurrence locations and sampled background conditions, rather than between confirmed presences and absences. Following the grading criteria used in this study, AUC values of 0.50–0.60, 0.60–0.70, 0.70–0.80, 0.80–0.90, and 0.90–1.00 were interpreted as failing, poor, fair, good, and excellent, respectively [42]. TSS was calculated by treating background points as pseudo-absences and was therefore interpreted as a complementary presence-background metric [43]. Jackknife tests were used to assess the relative importance of individual predictors. Model fitting and post-processing were conducted in R v4.5.3 using the dismo v1.3.16 [44] and pROC v1.19.0.1 [45] packages.

To provide a spatially more stringent assessment of model transferability, we additionally conducted four-fold spatial block cross-validation. Occurrence and background points were assigned to 250 km spatial blocks, which were grouped into four folds while maintaining occurrence records across folds as evenly as possible. In each iteration, one fold was withheld for validation, and background points in the withheld blocks were excluded from model calibration. Models were refitted using the ENMeval-selected RM–FC settings, and validation AUC was calculated from withheld occurrence records and background points. Fold-specific binary predictions were generated using MaxTSS thresholds derived from the corresponding training data. We then calculated background-based precision, recall, and F1-score from fold-specific confusion matrices: precision = TP/(TP + FP), recall = TP/(TP + FN), and F1-score = 2 × precision × recall/(precision + recall). Because true absence records were unavailable, false-positive and true-negative counts were derived from withheld background points. Therefore, confusion matrices, precision, recall, and F1-score were interpreted as presence-background validation metrics rather than conventional presence-absence diagnostic measures. Spatial block cross-validation was implemented in R v4.5.3 using terra v1.9.11, sf v1.1.1, maxnet v0.1.4, and pROC v1.19.0.1.

#### 2.4.3. Systematic Parameter Tuning for MaxEnt Modeling

The complexity and regularization level of MaxEnt are primarily controlled by two parameters: the regularization multiplier (RM) and the feature-class (FC) combination [11]. The model may employ five fundamental feature functions: linear (L), quadratic (Q), hinge (H), product (P), and threshold (T). To reduce overfitting and improve transferability, the optimal RM–FC combination was selected independently for each species using ENMeval v2.0.5.2 in R v4.5.3 [12]. The inputs to ENMeval included the spatially thinned occurrence records for each species, the 16 retained environmental predictors, and 10,000 background points generated within the Xinjiang administrative boundary.

A systematic grid-search design was used. The RM was tested at eight levels from 0.5 to 4.0 with an interval of 0.5, and six FC combinations were evaluated: L, H, LQ, LQH, LQHP, and LQHPT. This resulted in 48 candidate parameter configurations for each species. Each candidate model was evaluated within a five-fold cross-validation framework. Model performance under each parameter setting was assessed using the corrected Akaike Information Criterion (AICc) [46]. The relative fit of each candidate model to the optimal model was quantified as ΔAICc, calculated by subtracting the minimum AICc value among all candidate models from the AICc of each model. According to established criteria, models with ΔAICc < 2 are considered to have substantial statistical support, while those with ΔAICc > 10 are considered to have essentially no support [47]. For each species, the configuration yielding the minimum AICc, corresponding to ΔAICc = 0, was selected as the final parameterization. The specific optimization results and associated predictive performance metrics are reported in Section 3.1.

#### 2.4.4. Classification and Analysis of Prediction Results

Continuous MaxEnt logistic outputs were mapped in ArcGIS Pro 3.0.2 and displayed as four suitability classes: unsuitable (0–0.1), low (0.1–0.3), medium (0.3–0.5), and high (0.5–1.0) [48,49]. These classes were used for visualization only. For change analyses, continuous predictions were converted to binary suitable/unsuitable maps using species-specific thresholds that maximized training sensitivity plus specificity (hereafter, MaxTSS thresholds), which are equivalent to thresholds maximizing the true skill statistic [50]. Current and future binary maps were overlaid to classify each grid cell as stable unsuitable, expansion (newly suitable), contraction (newly unsuitable), or stable suitable. Areas of each category were calculated for each species, future period, and SSP scenario. Because the primary analysis indicated negligible contraction for *A. camelorum* and *G. inflata*, we assessed threshold sensitivity for these species. In addition to MaxTSS, binary maps were generated using the 10th-percentile training-presence and equal sensitivity–specificity thresholds. Expansion, contraction, stable suitable area, and net change were compared among threshold methods (Table S11).

To characterize environmental conditions associated with projected habitat change, values of the dominant predictor were extracted from expansion, contraction, and stable suitable cells for each scenario and future period. Elevation was used for *A. camelorum* (permutation importance = 27.6%), precipitation seasonality (Bio15) for *T. arcuata* (52.2%), and minimum temperature of the coldest month (Bio6) for *G. inflata* (58.5%). Distributions were visualized using violin plots with embedded boxplots; sample sizes represent the number of 30 arc-second grid cells. These analyses were conducted in R v4.5.3 using terra v1.9.11 and ggplot2 v4.0.2. We further evaluated whether projected habitat changes were concentrated along elevation and soil-salinity gradients. Binary change maps were overlaid with elevation and soil electrical conductivity (ECE), the latter used as a proxy for soil salinity. Elevation was classified as <500, 500–1000, 1000–2000, and >2000 m, whereas ECE was classified as <2, 2–4, 4–8, and >8 dS m^−1^, representing non- or weakly saline, slightly saline, moderately saline, and strongly saline soils, respectively. For each species, scenario, and future period, we calculated the area and proportion of each habitat-change category within each elevation and salinity class (Tables S7 and S8).

#### 2.4.5. Niche Breadth and Overlap Analysis

Niche breadth for each species was quantified using Levins’ *B*1 and *B*2 indices [51]. For each environmental variable, the frequency distribution of species occurrences across equally spaced bins was calculated, and B1 was computed as (Equation (3)):

(3) $$B_{1}=\frac{1}{n}\sum_{j=1}^{n}\frac{1}{\sum_{i=1}^{k}p_{ij}^{2}}$$

where *p*_ij_ is the proportion of occurrence records in the *i*-th bin of the *j*-th environmental variable, *k* is the number of bins, and *n* is the total number of environmental variables. *B*2 normalizes *B*1 to a 0–1 range (Equation (4)):

(4) $$B_{2}=\frac{B_{1}-1}{k_{avg}-1}$$

where *k_avg_* is the average number of bins across all variables. Higher *B*2 values indicate wider ecological tolerance. Pairwise niche overlap was assessed using Schoener’s D [52] and Hellinger’s I [53], both implemented using a grid-based density algorithm in a PCA-transformed environmental space. Prior to PCA, the 16 retained environmental predictors were standardized to zero mean and unit variance to remove the effects of different measurement units. PCA was then performed using the environmental background of Xinjiang together with the occurrence records of the three focal species, so that all species were projected into a common environmental space. Principal components were ranked according to their explained variance. The first two axes, PC1 and PC2, were retained because they explained the largest proportions of total environmental variation and provided the two-dimensional environmental space required by the PCA-env niche overlap framework. In this study, PC1 explained 29.3% of the total environmental variance and PC2 explained 20.9%, with a cumulative explained variance of 50.2%. The same PCA space was used for all pairwise comparisons to ensure that Schoener’s D and Hellinger’s I were calculated under a consistent environmental background. For two species with probability density vectors p and q in this gridded environmental space (grid resolution = 80 × 80 cells), Schoener’s D and Hellinger’s I were calculated as follows (Equations (5) and (6)):

(5) $$D=1-\frac{1}{2}\sum_{i=1}^{m}|p_{i}-q_{i}|$$

(6) $$I=1-\frac{1}{2}\sqrt{\sum_{i=1}^{m}{\left(\sqrt{p_{i}}-\sqrt{q_{i}}\right)}^{2}}$$

where *m* is the total number of grid cells. Both indices range from 0 (no overlap) to 1 (complete overlap). All analyses were conducted using the 16 final environmental predictors retained in the MaxEnt models, with 5000 random background points sampled from within the Xinjiang administrative boundary to characterize the available environmental space. Calculations were performed in R v4.5.3 using ecospat v4.1.3 [54] and custom R scripts for Levins’ B indices.

#### 2.4.6. Statistical Uncertainty and Randomization Tests

To provide quantitative support for species-level comparisons, we conducted bootstrap and randomization tests for niche breadth, niche overlap, and projected habitat-change metrics. These analyses were not designed to test life-form effects, because each life-form category was represented by only one species.

For niche breadth, occurrence records for each species were resampled with replacement 999 times. Levins’ standardized B2 was recalculated for each replicate using environmental-variable bins defined from the background environment. Bootstrap 95% confidence intervals were derived from the 2.5th and 97.5th percentiles. This procedure quantified uncertainty in niche-breadth estimates, particularly for *T. arcuata*, which had the fewest valid occurrence records. Pairwise differences in B2 were calculated across bootstrap replicates and considered statistically supported when their 95% confidence intervals excluded zero. Pairwise Schoener’s D and Hellinger’s I were similarly recalculated across 999 bootstrap replicates by independently resampling occurrence records for each species. Niche-equivalency tests were additionally performed by pooling records for each species pair and randomly reallocating species labels while retaining the original sample sizes. Observed overlap values were then compared with randomized null distributions to assess whether they differed from expectations under niche equivalency.

For projected habitat-change metrics, uncertainty was estimated using 100 bootstrap SDM projections per species. Occurrence records were resampled with replacement, and MaxEnt models were refitted using the ENMeval-selected RM and FC settings. Current and future binary suitability maps were generated using replicate-specific MaxTSS thresholds, from which expansion, contraction, stable suitable area, and net change were recalculated for each period and SSP scenario. Pairwise differences in projected net change were evaluated using bootstrap confidence intervals. These comparisons were interpreted as species-specific model outcomes, rather than formal tests of life-form effects.

## 3. Results

### 3.1. Optimization and Predictive Performance Evaluation

**Figure 3:**
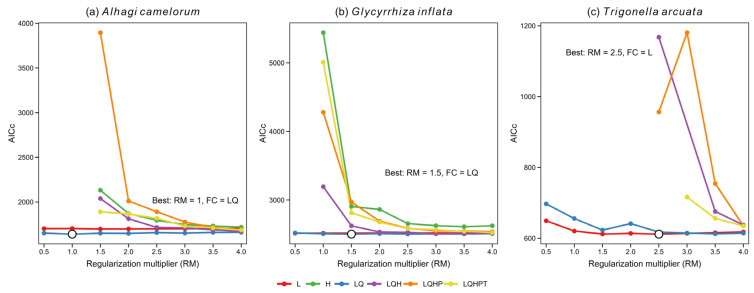
MaxEnt parameter optimization results for the three legume species—grid search over the regularization multiplier (RM) and feature class (FC) combinations, with the optimal RM–FC configurations highlighted for: (**a**) *Alhagi camelorum*; (**b**) *Glycyrrhiza inflata*; (**c**) *Trigonella arcuata*.

**Figure 4:**
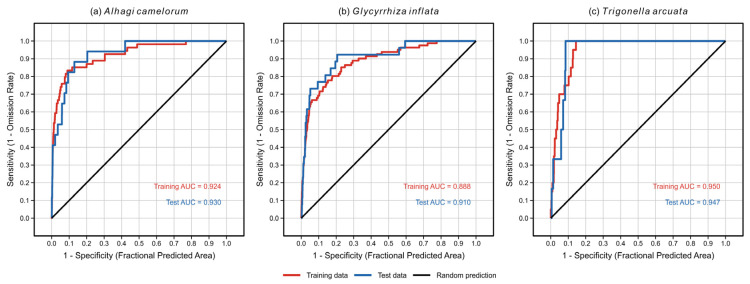
Receiver operating characteristic (ROC) curves and area under the curve (AUC) for the three legume species: (**a**) *Alhagi camelorum*; (**b**) *Glycyrrhiza inflata*; (**c**) *Trigonella arcuata*. The red curve represents the training data, and the blue curve represents the test data.

ENMeval evaluated 48 parameterizations per species, combining eight regularization multipliers (RM = 0.5–4.0, in increments of 0.5) with six feature-class (FC) combinations (L, H, LQ, LQH, LQHP, and LQHPT). Optimal models were selected using the minimum AICc criterion (ΔAICc = 0), which balances model fit and complexity [12] (Figure 3). The optimal settings were RM = 1 and FC = LQ for *A. camelorum*, RM = 1.5 and FC = LQ for *G. inflata*, and RM = 2.5 and FC = L for *T. arcuata*. Test AUC values were 0.930, 0.910, and 0.947, respectively, and MaxTSS values were 0.778, 0.691, and 0.870 (Figure 4). These metrics indicated good apparent discriminatory performance within the Xinjiang-wide calibration domain. The selected LQ models for *A. camelorum* and *G. inflata* represented relatively simple nonlinear responses, whereas the more strongly regularized linear model for *T. arcuata* was consistent with its smaller occurrence dataset and narrower sampled environmental range. These parameter differences were interpreted as species-specific modeling patterns rather than evidence of life-form effects.

The *T. arcuata* model requires particular caution because only 29 spatially thinned occurrence records were available. Although the primary model showed high apparent performance, test metrics may be inflated when occurrence records are few or occupy a restricted environmental space. The restricted-domain sensitivity analysis further showed that predictions were sensitive to background extent. Within a 100 km buffer around occurrence records, the model selected RM = 4 and FC = LQHP, with an apparent AUC of 0.813, MaxTSS of 0.509, and mean cross-validation AUC of 0.713 (Table S9). Accordingly, projections for *T. arcuata* were treated as exploratory estimates of potential suitability conditional on the selected calibration domain.

The NDVI-exclusion analysis indicated that the temporal mismatch between NDVI and baseline climatic data most strongly affected *A. camelorum*. Removing NDVI reduced AUC and MaxTSS from 0.930 to 0.904 and from 0.778 to 0.688, respectively, for *A. camelorum*. Corresponding values declined from 0.910 to 0.887 and from 0.691 to 0.611 for *G. inflata*, whereas *T. arcuata* showed little change (AUC = 0.949; MaxTSS = 0.850). Predictions for *A. camelorum* should therefore be interpreted as conditional on the recent vegetation-productivity layer included in the full model (Table S10).

Post-selection spatial-block cross-validation provided a more spatially stringent, although not fully nested, assessment of transferability. Mean validation AUC values were 0.913 ± 0.043 for *A. camelorum*, 0.834 ± 0.097 for *G. inflata*, and 0.914 ± 0.080 for *T. arcuata*. Mean TSS values were 0.677 ± 0.129, 0.523 ± 0.215, and 0.644 ± 0.252, respectively. Mean recall was 0.787 ± 0.130 for *A. camelorum*, 0.665 ± 0.201 for *G. inflata*, and 0.762 ± 0.210 for *T. arcuata*. Lower precision values reflected the use of withheld background points rather than confirmed absences. Greater among-fold variation in TSS for *G. inflata* and *T. arcuata* indicated greater uncertainty in spatial transferability (Tables S12 and S13).

Occurrence records of all species were associated with higher sampling-effort values than expected under the sampling-effort null model (*A. camelorum*, *p* = 0.001; *G. inflata*, *p* = 0.001; *T. arcuata*, *p* = 0.002), although their observed spatial clustering did not exceed null expectations. Sampling-bias correction reduced AUC by 0.127, 0.149, and 0.042 for *A. camelorum*, *G. inflata*, and *T. arcuata*, respectively, and altered estimated current suitable area by −61.3%, +94.0%, and +33.5%. Suitable-area estimates, particularly for *A. camelorum* and *G. inflata*, should therefore be interpreted as conditional on regional collection effort (Table S14).

### 3.2. Present-Day Suitability Patterns of the Three Legume Species

**Figure 5:**
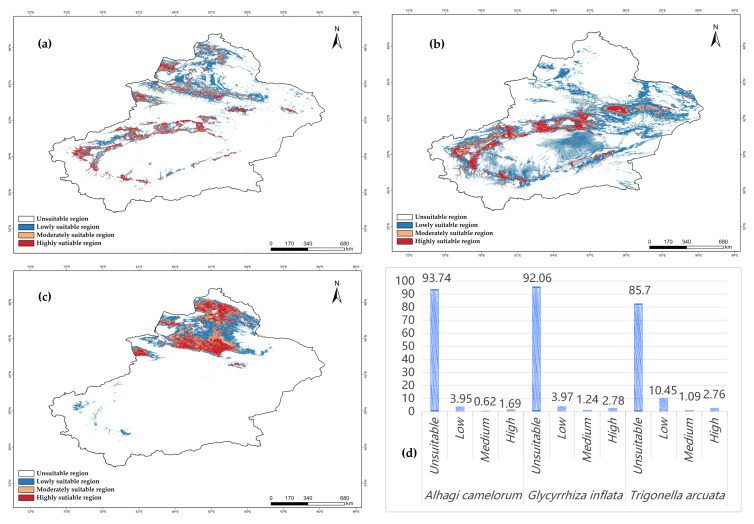
Present-day habitat suitability for the three legume species. (**a**–**c**) Spatial patterns of suitability classes for (**a**) *Alhagi camelorum*, (**b**) *Glycyrrhiza inflata*, and (**c**) *Trigonella arcuata*. (**d**) Proportions of suitability classes within the total area of Xinjiang for each species. The administrative boundaries of the Xinjiang Uygur Autonomous Region were derived from the standard map downloaded from the Standard Map Service of the Ministry of Natural Resources of the People’s Republic of China (review number GS(2024)0650), and the base-map boundaries were not modified.

Present-day habitat suitability differed markedly among the three species (Figure 5). *Alhagi camelorum* showed a dispersed trans-Tianshan pattern, with suitable areas distributed across piedmont oases, alluvial fans, and basin margins in both northern and southern Xinjiang. *Glycyrrhiza inflata* formed the most continuous suitability core along the northern Tarim Basin oasis belt, whereas *Trigonella arcuata* showed a fragmented northern distribution concentrated along the southern Junggar Basin and northern Tianshan piedmont plains. Across species, high- and medium-suitability areas were associated mainly with oasis systems, river corridors, alluvial fans, and piedmont zones, whereas the hyper-arid interiors of the Taklamakan and Gurbantunggut deserts were predominantly unsuitable. Thus, the species shared a general association with oasis–piedmont environments but differed in geographic center, spatial continuity, and fragmentation.

### 3.3. Niche Breadth and Overlap Among Species

Bootstrap analyses quantified uncertainty in niche-breadth estimates, particularly for *T. arcuata*, which had the smallest occurrence dataset. *A. camelorum* had an observed Levins’ B2 of 0.329 (95% CI: 0.284–0.339), comparable to that of *G. inflata* (B2 = 0.315; 95% CI: 0.274–0.330). *T. arcuata* had the narrowest niche breadth (B2 = 0.225; 95% CI: 0.162–0.241; Table 2). The bootstrap difference between *A. camelorum* and *G. inflata* was not statistically supported (ΔB2 = 0.014; 95% CI: −0.031 to 0.049). By contrast, both species had significantly broader niches than *T. arcuata*, with pairwise B2 differences of 0.104 and 0.090, respectively (Table S15).

Niche overlap was generally low to moderate. The highest overlap occurred between *A. camelorum* and *G. inflata* (Schoener’s D = 0.234; Hellinger’s I = 0.296), followed by *A. camelorum* and *T. arcuata* (D = 0.056; I = 0.093). Overlap was lowest between *G. inflata* and *T. arcuata* (D = 0.019; I = 0.038; Table 3). Although these estimates indicate partial separation in environmental-space occupancy, two-sided niche-equivalency tests did not reject the null hypothesis of equivalency for any species pair (α = 0.05). Bootstrap uncertainty intervals likewise indicated limited overlap among species pairs (Table S16). Accordingly, the observed differences in niche breadth and overlap should not be interpreted as conclusive evidence of niche non-equivalency.

To further characterize the environmental gradients underlying niche patterns, we examined PCA loadings for the 16 retained predictors in the two-dimensional environmental space used for niche-overlap analyses. PC1 explained 29.3% of total environmental variance and was primarily associated with warm-season precipitation, soil organic carbon, winter temperature, mean diurnal temperature range, soil bulk density, terrain ruggedness, and elevation. PC2 explained 20.9% of the variance and was mainly associated with precipitation seasonality, soil pH, clay content, elevation, cold-season precipitation, and NDVI. Together, the first two axes explained 50.2% of the environmental variance and defined the two-dimensional space used for PCA-env overlap analyses. The overlap estimates therefore represent dominant, rather than all multidimensional, environmental gradients. These loadings further indicate that the species differed in the environmental gradients associated with their niche positions, in addition to differences in niche breadth and overlap estimates (Table S6).

### 3.4. Contributions of Environmental Predictors

**Figure 6:**
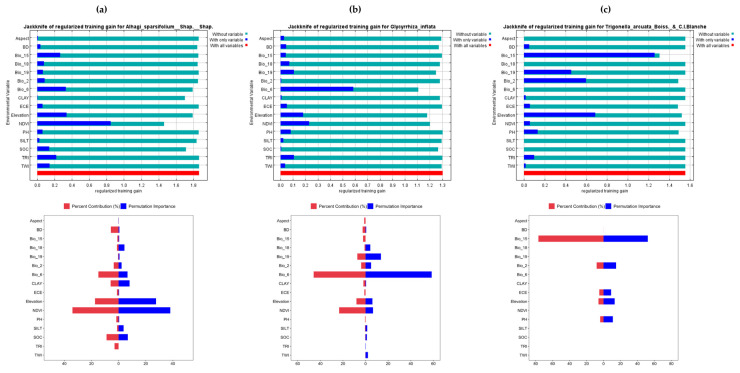
Jackknife tests and variable-importance metrics for the three legume species: (**a**) *Alhagi camelorum*; (**b**) *Glycyrrhiza inflata*; and (**c**) *Trigonella arcuata*. In each panel, the upper plot shows the jackknife test based on regularized training gain, where cyan bars represent model gain when the corresponding variable was excluded, blue bars represent model gain when the variable was used in isolation, and the red bar represents model gain when all variables were included. The lower plot compares the percent contribution (red) and permutation importance (blue) of each environmental variable.

**Figure 7:**
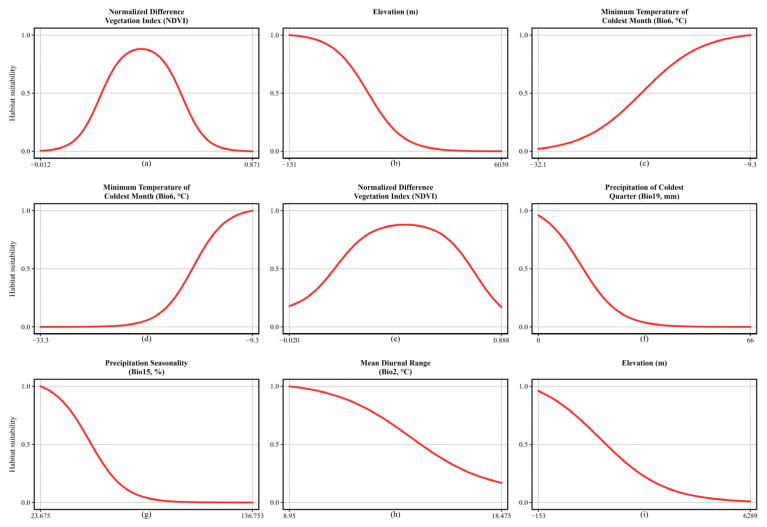
Response curves of the three most influential environmental variables for the three legume species: (**a**–**c**) *Alhagi camelorum*: (**a**) normalized difference vegetation index (NDVI), (**b**) elevation, and (**c**) minimum temperature of the coldest month (Bio6); (**d**–**f**) *Glycyrrhiza inflata*: (**d**) minimum temperature of the coldest month (Bio6), (**e**) normalized difference vegetation index (NDVI), and (**f**) precipitation of the coldest quarter (Bio19); and (**g**–**i**) *Trigonella arcuata*: (**g**) precipitation seasonality (Bio15), (**h**) mean diurnal range (Bio2), and (**i**) elevation.

Although the same non-collinear predictor framework was used for comparative modeling, the three species showed clearly different responses to environmental factors, as indicated by variable contributions, permutation importance, jackknife tests, and response curves. The potential distributions of the three Fabaceae species in Xinjiang were shaped by distinct combinations of climatic, vegetation, and topographic predictors (Figure 6). For *Alhagi camelorum*, habitat suitability was mainly associated with annual maximum NDVI (34.0%) and elevation (17.4%), which together explained 51.4% of model contribution. Minimum temperature of the coldest month, Bio6, provided additional explanatory power (14.9%). Permutation importance and jackknife tests also identified NDVI and elevation as the strongest independent predictors. Response curves showed that suitability peaked at moderate NDVI values (0.35–0.40) and at low elevations, from below sea level to approximately 500 m (Figure 7a,b). Suitability declined sharply at high NDVI values and decreased almost linearly above 500 m. For Bio6, suitability was close to zero below −35 °C but increased steeply above −10 °C (Figure 7c). These patterns are consistent with the occurrence of *A. camelorum* in low-elevation oases and alluvial plains, where moderate vegetation cover, relatively mild winters, and low-relief terrain provide favorable conditions [36]. However, because the NDVI layer represents 2000–2024 vegetation productivity whereas the bioclimatic predictors represent 1970–2000 climate normals, NDVI-associated responses for *A. camelorum* should be interpreted as associations with recent vegetation-productivity conditions rather than strictly synchronous climate–vegetation relationships.

For *Glycyrrhiza inflata*, Bio6 was the dominant predictor, accounting for 45.9% of percent contribution and 58.5% of permutation importance. NDVI (23.4%), elevation (8.1%), and precipitation of the coldest quarter, Bio19 (7.3%), had secondary effects. Suitability was highest when Bio6 ranged from −12 °C to −5 °C but dropped sharply below −30 °C (Figure 7d). NDVI showed a broad unimodal response, with peak suitability at 0.60–0.85, indicating a relatively high tolerance of dense vegetation cover (Figure 7e). The Bio19 model shows that its suitability declines rapidly as winter precipitation increases, indicating a narrow optimal range (Figure 7f). This winter-temperature-dominated response, together with sensitivity to cold-season moisture, matches the concentration of *G. inflata* in the northern Tarim Basin oasis belt. It also suggests that reduced winter cold stress may be a key factor underlying its projected expansion under future warming.

For *T. arcuata*, Bio15 had the highest percent contribution (76.8%), whereas Bio2 showed the highest permutation importance, indicating that precipitation seasonality strongly influenced model fitting while diurnal temperature range contributed substantial independent discriminatory information when permuted. Response curves indicated that suitability was highest when precipitation seasonality was low to moderate (10–25%) and declined rapidly above this range (Figure 7g). Bio2 showed a narrow optimum of 8–10 °C, with suitability falling sharply above 15 °C and approaching zero above 18 °C (Figure 7h). Elevation effects were also evident: suitability was highest at very low elevations and declined steadily with increasing altitude (Figure 7i). These results indicate that *T. arcuata* is associated with low-elevation habitats characterized by relatively even precipitation and limited diurnal temperature variation, consistent with its restriction to the piedmont plains of northern Xinjiang.

### 3.5. Potential Habitat Changes Under Future Climate Scenarios

**Figure 8:**
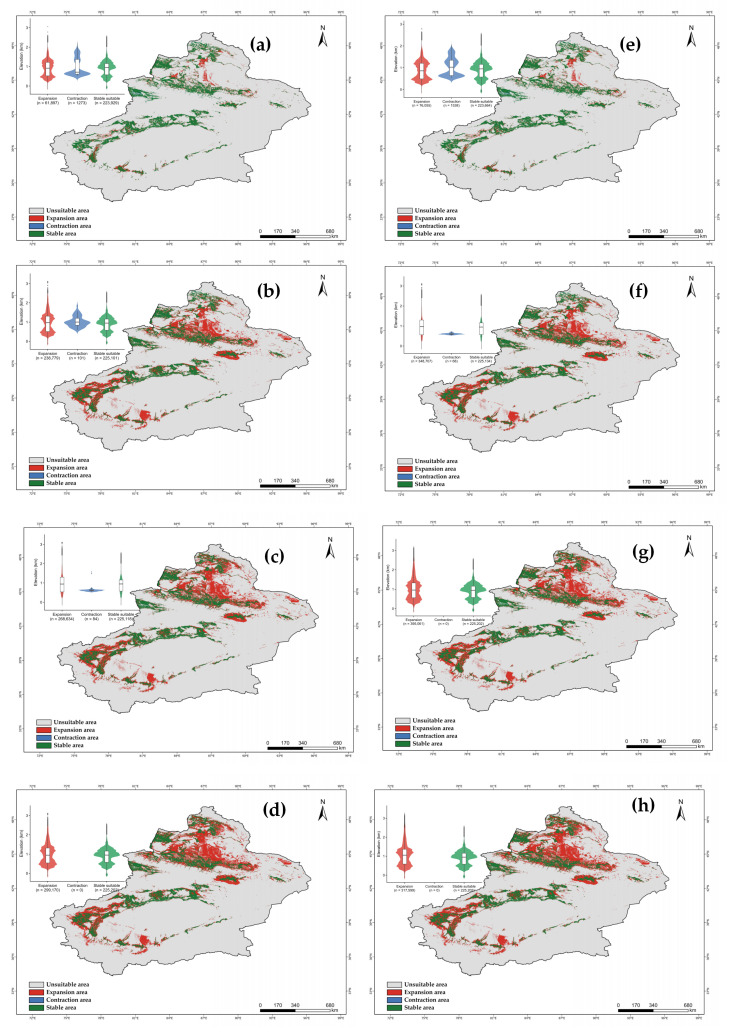
Projected habitat changes and elevational signatures of expansion and contraction zones for *Alhagi camelorum* under four SSP scenarios for 2061–2080 (left panels, (**a**–**d**)) and 2081–2100 (right panels, (**e**–**h**)). Maps show habitat change categories: stable unsuitable (grey), expansion (red), contraction (blue), and stable suitable (green). Embedded violin plots with boxplots show the distribution of Elevation within expansion, contraction, and stable suitable zones for each corresponding scenario. Boxplots indicate median (center line), interquartile range (box), and 1.5× IQR (whiskers). Sample sizes (n) denote the number of 30 arc-second grid cells in each zone. The administrative boundaries of the Xinjiang Uygur Autonomous Region were derived from the standard map downloaded from the Standard Map Service of the Ministry of Natural Resources of the People’s Republic of China (review number GS(2024)0650), and the base-map boundaries were not modified.

**Figure 9:**
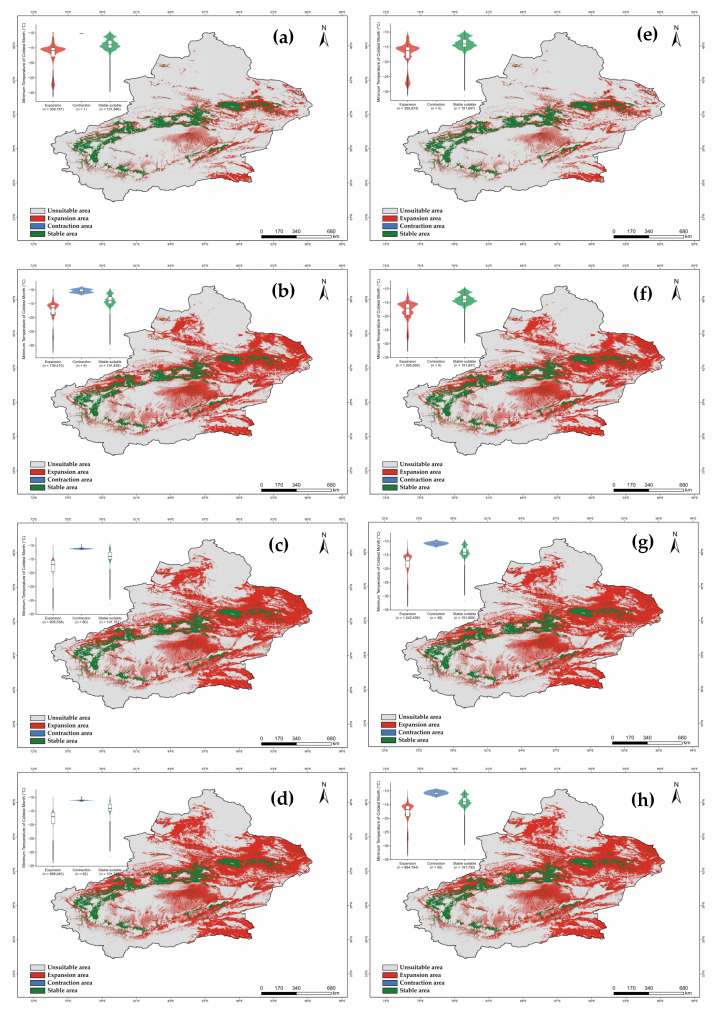
Projected habitat changes and winter temperature signatures of expansion and contraction zones for *Glycyrrhiza inflata* under four SSP scenarios for 2061–2080 (left panels, (**a**–**d**)) and 2081–2100 (right panels, (**e**–**h**)). Maps show habitat change categories: stable unsuitable (grey), expansion (red), contraction (blue), and stable suitable (green). Embedded violin plots with boxplots show the distribution of minimum temperature of the coldest month (Bio6) within expansion, contraction, and stable suitable zones for each corresponding scenario. Boxplots indicate median (center line), interquartile range (box), and 1.5× IQR (whiskers). Sample sizes (n) denote the number of 30 arc-second grid cells in each zone. The administrative boundaries of the Xinjiang Uygur Autonomous Region were derived from the standard map downloaded from the Standard Map Service of the Ministry of Natural Resources of the People’s Republic of China (review number GS(2024)0650), and the base-map boundaries were not modified.

**Figure 10:**
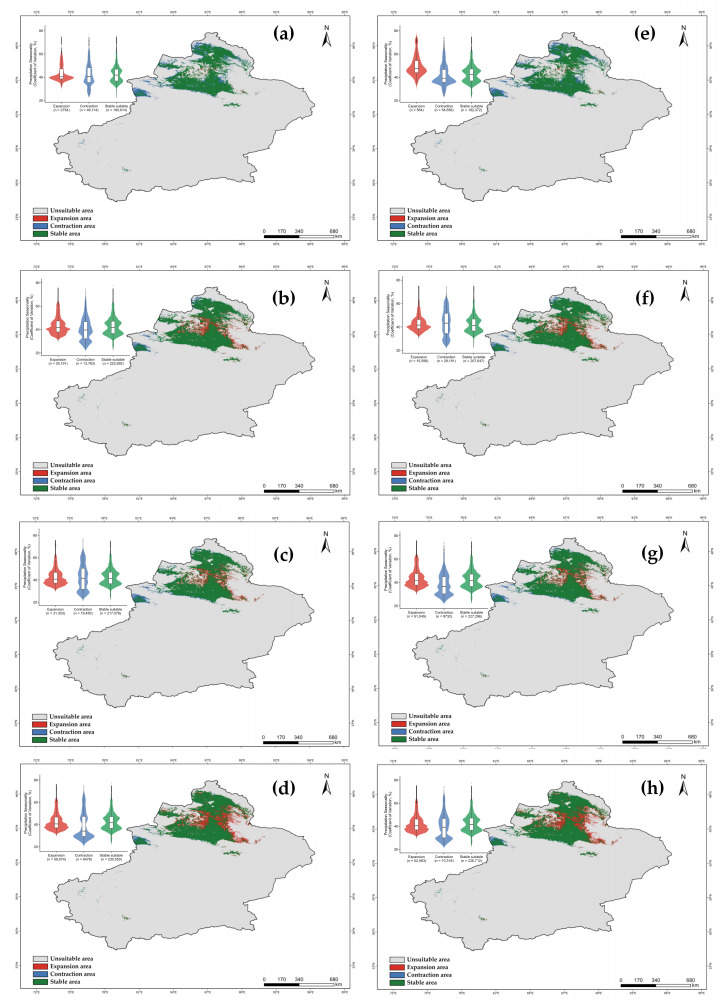
Projected habitat changes and precipitation seasonality signatures of expansion and contraction zones for *Trigonella arcuata* under four SSP scenarios for 2061–2080 (left panels, (**a**–**d**)) and 2081–2100 (right panels, (**e**–**h**)). Maps show habitat change categories: stable unsuitable (grey), expansion (red), contraction (blue), and stable suitable (green). Embedded violin plots with boxplots show the distribution of precipitation seasonality (Bio15) within expansion, contraction, and stable suitable zones for each corresponding scenario. Boxplots indicate median (center line), interquartile range (box), and 1.5× IQR (whiskers). Sample sizes (n) denote the number of 30 arc-second grid cells in each zone. The administrative boundaries of the Xinjiang Uygur Autonomous Region were derived from the standard map downloaded from the Standard Map Service of the Ministry of Natural Resources of the People’s Republic of China (review number GS(2024)0650), and the base-map boundaries were not modified.

Future projections revealed contrasting, species-specific changes in potential habitat suitability under the four SSP scenarios (Table 4; Figure 8, Figure 9 and Figure 10). *Alhagi camelorum* and *Glycyrrhiza inflata* showed predominantly expansion-driven changes, whereas *Trigonella arcuata* exhibited stronger scenario- and period-dependent variation. Expansion generally occurred along the margins of currently suitable habitats. For *A. camelorum*, newly suitable areas were concentrated along piedmont plains, basin margins, and oasis–desert transition zones. Expansion of *G. inflata* extended outward from its current northern Tarim Basin core, whereas projected changes in *T. arcuata* were concentrated around the Junggar Basin and northern Tianshan piedmont plains. Stratified analyses showed that expansion of *A. camelorum* and *G. inflata* occurred mainly at elevations of 500–2000 m and in non-saline or weakly saline soils. Changes in *T. arcuata* were concentrated primarily below 1000 m and in low-salinity environments (Tables S7 and S8).

Bootstrap projections indicated substantial interspecific differences in net change (Table S17). *G. inflata* showed the largest mean gains, ranging from 2037.0 to 6055.9 × 10^2^ km^2^, whereas *A. camelorum* showed consistently positive gains of 316.9–1309.9 × 10^2^ km^2^. *T. arcuata* showed weaker and more uncertain responses, including significant losses under SSP1-2.6 in both periods (−171.8 and −183.1 × 10^2^ km^2^); confidence intervals generally overlapped zero under the other scenarios. Pairwise bootstrap comparisons supported differences among all three species, with *G. inflata* showing the largest gains, followed by *A. camelorum* and *T. arcuata*.

Threshold-sensitivity analyses using the 10th-percentile training-presence and equal sensitivity–specificity thresholds retained the expansion-dominated patterns for *A. camelorum* and *G. inflata*. Their limited contraction was therefore not attributable solely to the MaxTSS threshold, although these projections remain conditional on model structure and predictor uncertainty (Table S11).

## 4. Discussion

### 4.1. Environmental Correlates of Current Habitat Suitability

The environmental associations identified in this study were broadly consistent with previous ecological research, while revealing marked species- and scale-dependent differences. For *Alhagi camelorum*, its association with NDVI and low-elevation habitats accords with studies of Xinjiang camelthorn populations, particularly *A. camelorum*, which describe a deep-rooted phreatophytic strategy and strong groundwater dependence in oasis-desert transition zones [27,55]. NDVI likely served as a spatial proxy for vegetation productivity and, potentially, the hydrological integrity of oasis–riparian systems; however, the correlative model used here cannot distinguish these mechanisms directly. The weak regional contribution of soil salinity should not be interpreted as evidence that salinity is unimportant. Rather, it may reflect the relatively broad stress tolerance of camelthorn or covariance among salinity, groundwater, and landform conditions.

For *Glycyrrhiza inflata*, the importance of climatic predictors is consistent with previous MaxEnt studies. Earlier China-wide analyses emphasized precipitation-related predictors [18], whereas winter minimum temperature was more influential in our Xinjiang-scale model. This difference likely reflects variation in spatial extent, occurrence coverage, background selection, and the environmental gradients represented in each study. Population-level research has also shown that water availability and habitat fragmentation influence the spatial structure of *G. inflata* [27]. Together, these findings suggest that its distribution is jointly constrained by thermal conditions and oasis hydrology, with their relative importance varying across spatial scales.

Direct distribution modeling of *Trigonella arcuata* remains limited. However, experimental studies of Central Asian desert ephemerals show that reduced precipitation decreases survival, reproduction, seed production, and biomass [56]. This evidence is consistent with the strong association between *T. arcuata* suitability and precipitation seasonality in our model. Because annual species must complete germination, growth, and reproduction within a short favorable period, irregular precipitation may restrict suitable establishment windows. The modeled importance of diurnal temperature range represents a plausible additional constraint but remains a testable hypothesis because direct physiological evidence for *T. arcuata* is lacking.

Overall, these results support previous evidence that dryland plant distributions are jointly shaped by climatic and hydrological constraints, while showing that the dominant environmental filters differ among species and across spatial scales. These contrasts should therefore be interpreted primarily as species-specific responses, rather than as definitive evidence of life-form effects.

### 4.2. Projected Changes in Potential Habitat Suitability Under Future Scenarios

The divergent projections indicate that warming does not elicit uniform responses among the three dryland legumes but instead interacts with species-specific environmental constraints [1]. The large projected gains of *G. inflata* are consistent with a release from winter temperature limitations. Earlier studies likewise identified climate as a major determinant of its distribution, although the relative importance of temperature and precipitation varies across spatial scales [18,57]. *Alhagi camelorum* showed a different pattern. Its moderate but persistent gains, together with its relatively broad niche, suggest that tolerance of heterogeneous oasis–piedmont environments may buffer its response to climate change without necessarily producing the largest projected expansion. This interpretation is consistent with the drought-adapted and phreatophytic ecology reported for camelthorn populations in Xinjiang [27].

In contrast, the response of *T. arcuata* was strongly scenario dependent. As an annual species, its establishment and reproduction depend on suitable seasonal precipitation and thermal windows; shifts in precipitation seasonality and diurnal temperature range may therefore limit stable expansion, even where warming increases potential suitability. Given its small occurrence dataset and substantial sensitivity to background-domain definition, this proposed mechanism should be regarded as a species-specific hypothesis rather than a general response of annual herbs.

Overall, these contrasts are compatible with differences in rooting strategy, perenniality, and regeneration mode. They cannot, however, be statistically attributed to life form, because each category was represented by only one species. Species identity, evolutionary history, local adaptation, dispersal capacity, and sampling structure may also contribute to the observed patterns [58].

### 4.3. Uncertainty and Implications of Future Habitat Predictions

The principal uncertainty concerns *T. arcuata*, for which only 29 spatially thinned records were available [59,60]. Although its optimized model was simple and strongly regularized, the restricted-domain analysis reduced apparent AUC from 0.947 to 0.813 and MaxTSS from 0.870 to 0.509. Its projections should therefore be regarded as exploratory estimates, despite bootstrap evidence that it retained the narrowest niche breadth (B2 = 0.225; 95% CI: 0.162–0.241). Because RM–FC settings were selected using the full occurrence dataset before spatial-block validation, the resulting validation metrics should be interpreted as post-selection estimates of transferability, rather than fully nested independent validation. Additional independent occurrence records and external validation are required.

Residual sampling bias also affected model outputs. Although records were standardized, screened, and spatially thinned, bias correction substantially altered performance and estimated suitable area, particularly for *A. camelorum* and *G. inflata*. The predictions therefore represent potential suitability conditional on the available occurrence data and their collection structure, rather than bias-free realized distributions.

Predictor uncertainty was greatest for NDVI, which represented vegetation productivity during 2000–2024, whereas climatic normals represented 1970–2000. Removing NDVI reduced the performance of the *A. camelorum* model from AUC = 0.930 and MaxTSS = 0.778 to 0.904 and 0.688, respectively. Its projections should, consequently, be interpreted as conditional on recent vegetation-productivity conditions [22,24].

Further uncertainty arises from threshold selection, spatial transferability, and the use of a single GCM. Threshold-sensitivity analyses indicated that the limited contraction projected for *A. camelorum* and *G. inflata* was not solely attributable to the MaxTSS threshold, whereas spatial-block validation showed greater instability for *G. inflata* and *T. arcuata*. Multi-GCM and multi-algorithm ensembles, dynamic predictors, and independent validation are therefore needed to strengthen future projections [36,61,62,63]. Moreover, dispersal, groundwater availability, grazing, land-use change, biotic interactions, and local adaptation were not modeled [64]. Projected suitability should thus not be interpreted as future occupancy, because dispersal limitation, extinction debt, and delayed colonization may prevent realized range shifts [65]. Nevertheless, these species-specific results provide a useful basis for differentiated conservation and restoration planning [66,67].

## 5. Conclusions

This study used ENMeval-optimized MaxEnt models to compare climate-associated habitat suitability among three dryland Fabaceae species in Xinjiang. The species differed in their dominant environmental correlates and projected suitability trajectories. *Alhagi camelorum* and *Glycyrrhiza inflata* exhibited expansion-dominated suitability patterns under most modeled scenarios, whereas *Trigonella arcuata* showed more variable and uncertain responses. Observed niche breadth was narrower for *T. arcuata*; however, niche-equivalency tests provided no conclusive evidence that the species occupied non-equivalent niches. These results should be interpreted as species-specific model outcomes, rather than evidence of life-form effects, because each life-form category was represented by only one species.

The projections nonetheless provide species-specific guidance for conservation planning. For *G. inflata*, projected gains were concentrated around the northern Tarim Basin oasis belt, where conservation should prioritize oasis–riparian connectivity. For *A. camelorum*, restoration efforts should focus on low-elevation oasis-desert transition zones while avoiding interpretation of NDVI-associated suitability as evidence of a direct physiological response. For *T. arcuata*, targeted occurrence surveys and monitoring in the Junggar Basin and northern Tianshan piedmont should precede spatially explicit conservation designation, given the sensitivity of its projections to sample size and background-domain assumptions. Future studies should incorporate broader taxonomic replication, independent occurrence data, biologically informed accessible areas, and multi-GCM projections to improve assessments of dryland community reassembly under climate change.

## Figures and Tables

**Table 1:** Environmental variables retained after screening and used for model construction for the three legume species.

Environmental Factor	Description	Unit
Bio2	Mean Diurnal Range (Mean of monthly (max temp—min temp))	°C
Bio6	Min Temperature of Coldest Month	°C
Bio15	Precipitation Seasonality (Coefficient of variation)	%
Bio18	Precipitation of Warmest Quarter	mm
Bio19	Precipitation of Coldest Quarter	mm
Elevation	Altitude	m
TRI	Terrain Ruggedness Index	m
Aspect	Orientation of the slope	—
TWI	Topographic Wetness Index	—
pH	Soil pH in H_2_O	—
SOC	Soil Organic Carbon	%
SILT	Silt Content	%
CLAY	Clay Content	%
BD	Soil Bulk Density	g cm^−3^
ECE	Soil Electrical Conductivity	dS m^−1^
NDVI	Multiyear mean of annual maximum NDVI values for 2000–2024	—

**Table 2:** Niche breadth of the three legume species.

Species	Levins’ B2	95% CI	Interpretation
*A. camelorum*	0.329	0.284–0.339	Broad niche
*G. inflata*	0.315	0.274–0.330	Broad niche
*T. arcuata*	0.225	0.162–0.241	Narrower niche

**Table 3:** Summary of pairwise niche overlap among the three legume species.

Species 1	Species 2	Schoener’s D	Hellinger’s I
*A. camelorum*	*G. inflata*	0.234	0.296
*A. camelorum*	*T. arcuata*	0.056	0.093
*T. arcuata*	*G. inflata*	0.019	0.038

Note: Detailed bootstrap confidence intervals and niche equivalency randomization tests for Schoener’s D and Hellinger’s I are provided in Table S16.

**Table 4:** Full-model projections of changes in suitable habitat area for the three legume species under different SSP scenarios. Area values are expressed as ×10^2^ km^2^.

Species	Period	Scenario	Expansion	Contraction	Stable Suitable Area	Net Change
*Alhagi camelorum*	2070s	SSP1-2.6	92.2	0.6	979.0	91.6
		SSP2-4.5	799.0	0.0	984.9	799.0
		SSP3-7.0	925.4	0.0	984.8	925.4
		SSP5-8.5	1073.7	0.0	985.2	1073.7
	2090s	SSP1-2.6	135.0	0.3	938.0	134.7
		SSP2-4.5	1277.0	0.0	945.0	1277.0
		SSP3-7.0	1497.0	0.0	945.4	1497.0
		SSP5-8.5	1090.8	0.0	945.4	1090.8
*Glycyrrhiza inflata*	2070s	SSP1-2.6	878.9	0.0	862.3	878.9
		SSP2-4.5	3064.1	0.0	862.1	3064.1
		SSP3-7.0	4008.8	0.0	861.4	4008.8
		SSP5-8.5	3948.2	0.0	861.6	3948.2
	2090s	SSP1-2.6	906.9	0.0	862.3	906.9
		SSP2-4.5	4613.9	0.0	862.3	4613.9
		SSP3-7.0	4887.6	0.0	861.5	4887.6
		SSP5-8.5	3872.2	0.0	861.3	3872.2
*Trigonella arcuata*	2070s	SSP1-2.6	1.0	98.8	936.9	−97.8
		SSP2-4.5	63.7	23.7	1140.0	40.0
		SSP3-7.0	48.4	43.5	1105.2	4.9
		SSP5-8.5	150.3	9.4	1182.1	140.9
	2090s	SSP1-2.6	0.0	111.7	883.5	−111.7
		SSP2-4.5	17.8	59.3	1049.6	−41.5
		SSP3-7.0	138.0	14.2	1163.8	133.8
		SSP5-8.5	135.0	18.5	1161.9	116.5

Note: Values of 0.0 indicate areas rounded to one decimal place and should not be interpreted as evidence of complete biological absence of contraction. Bootstrap uncertainty estimates and pairwise comparisons of projected net change are provided in Table S17.

## Data Availability

All links to input data are reported in the manuscript, and all output data are available upon request to the authors.

## Supplementary Materials

The following supporting information can be downloaded at: https://www.mdpi.com/article/10.3390/plants15152254/s1, Figure S1: Pearson correlation heatmap of environmental variables used in the MaxEnt models; Figure S2: Sampling-effort surface and final thinned occurrence records used for sampling-bias assessment; Table S1: Environmental variables for the three legume species; Table S2: Initial VIF values for the 33 candidate environmental predictors; Table S3: Stepwise elimination of variables with VIF > 10; Table S4: Final VIF values for the 16 retained environmental predictors; Table S5: Key functional traits of the three focal dryland Fabaceae species; Table S6: PCA loadings of the 16 retained environmental predictors on PC1 and PC2; Table S7: Stratified area statistics of habitat-change categories along elevation gradients; Table S8: Stratified area statistics of habitat-change categories along soil-salinity gradients; Table S9: Sensitivity analysis of *Trigonella arcuata* under Xinjiang-wide and restricted-domain background settings; Table S10: NDVI-exclusion sensitivity analysis for evaluating the effect of NDVI temporal mismatch on MaxEnt model performance; Table S11: Threshold-sensitivity analysis of projected habitat-change areas for *A. camelorum* and *G. inflata*; Table S12: Spatial block cross-validation performance of optimized MaxEnt models for the three dryland Fabaceae species; Table S13: Background-based confusion matrices from spatial block cross-validation; Table S14: Sampling-bias assessment and bias-file-equivalent sensitivity analysis for the three focal legume species; Table S15: Bootstrap confidence intervals and pairwise comparisons of niche breadth among the three focal legume species; Table S16: Bootstrap confidence intervals and randomization tests for niche overlap among the three focal legume species; Table S17: Bootstrap uncertainty and pairwise comparisons of projected net habitat-suitability change among the three focal legume species.
