# Supplementary material for: Optimized MaxEnt Models Predict Species-Specific Habitat Suitability Shifts for Three Dryland Fabaceae Species in Xinjiang
Source: Plants (Basel). 2026 Jul 23;15(15):2254. doi: 10.3390/plants15152254 (PMC13468077; doi:10.3390/plants15152254)
Supplement: Supplementary file 1 [file plants-15-02254-s001.zip › Supplementary Materials1.pdf]

Supplementary Materials:

Table s1. Environmental variables for the three legume species.

**Environmental factor**	**Description**	**Unit**	**Source**
Bio1	Annual Mean Temperature	°C	WorldClim v2.1
Bio2	Mean diurnal temperature range	°C	WorldClim v2.1
Bio3	Isothermality (Bio2/Bio7 × 100)	%	WorldClim v2.1
Bio4	Temperature seasonality (standard deviation × 100)	°C	WorldClim v2.1
Bio5	Maximum temperature of the warmest month	°C	WorldClim v2.1
Bio6	Minimum temperature of the coldest month	°C	WorldClim v2.1
Bio7	Temperature annual range (Bio5–Bio6)	°C	WorldClim v2.1
Bio8	Mean temperature of the wettest quarter	°C	WorldClim v2.1
Bio9	Mean temperature of the driest quarter	°C	WorldClim v2.1

Bio10	Mean temperature of the warmest quarter	°C	WorldClim v2.1
Bio11	Mean temperature of the coldest quarter	°C	WorldClim v2.1
Bio12	Annual precipitation	mm	WorldClim v2.1
Bio13	Precipitation of the wettest month	mm	WorldClim v2.1
Bio14	Precipitation of the driest month	mm	WorldClim v2.1
Bio15	Precipitation seasonality (coefficient of variation)	%	WorldClim v2.1
Bio16	Precipitation of the wettest quarter	mm	WorldClim v2.1
Bio17	Precipitation of the driest quarter	mm	WorldClim v2.1
Bio18	Precipitation of the warmest quarter	mm	WorldClim v2.1
Bio19	Precipitation of the coldest quarter	mm	WorldClim v2.1

Elevation	Elevation above sea level	m	SRTM DEM
Slope	Slope gradient	°	Derived from DEM
Aspect	Slope orientation	–	Derived from DEM
TRI	Terrain Ruggedness Index	m	Derived from DEM
Roughness	Topographic roughness	m	Derived from DEM
TWI	Topographic Wetness Index	–	Derived from DEM
pH(H_2O)	Soil pH (H_2O)	_	HWSD v1.2
SOC	Soil Organic Carbon	%	HWSD v1.2
SAND	Sand Content	%	HWSD v1.2
SILT	Silt Content	%	HWSD v1.2
CLAY	Clay Content	%	HWSD v1.2
BD	Soil Bulk Density	kg/dm^3	HWSD v1.2
ECE	Soil Electrical Conductivity	dS/m	HWSD v1.2
NDVI	Multi-year (2000–2024) mean of annual maximum NDVI	_	MODIS MOD13A3

Table S5. Key functional traits of the three focal dryland Fabaceae species.

Species	Life form	Longevity	Rooting / water-acquisition strategy	Regeneration strategy	Expected environmental sensitivity
Alhagi camelorum	Semi-shrub	Perennial	Deep-rooted, phreatophytic	Vegetative persistence and sexual reproduction	Drought, salinity, elevation, oasis/riparian productivity
Glycyrrhiza inflata	Herb	Perennial	Developed belowground organs	Vegetative persistence and regrowth	Winter temperature, soil moisture, oasis habitat
Trigonella arcuata	Herb	Annual	Shallow-rooted annual strategy	Seed regeneration	Precipitation seasonality, diurnal temperature range, establishment conditions

Table S6. PCA loadings of the 16 retained environmental predictors on PC1 and PC2

Predictor	PC1 loading	PC2 loading	Main environmental gradient
Bio15	0.032	-0.441	Precipitation seasonality
Bio18	0.380	0.022	Warm-season precipitation
Bio19	0.190	0.342	Cold-season precipitation
Bio2	-0.318	-0.247	Diurnal temperature range
Bio6	-0.333	0.208	Winter temperature
Elevation	0.286	-0.353	Topographic gradient
Aspect	0.005	-0.001	Topographic gradient
TRI	0.305	-0.124	Terrain ruggedness
TWI	-0.251	0.123	Topographic wetness
pH	-0.038	0.396	Soil acidity–alkalinity
SOC	0.368	-0.029	Soil organic carbon
SILT	0.255	0.193	Soil texture
CLAY	0.039	0.376	Soil texture
BD	-0.310	-0.004	Soil bulk density

ECE	-0.054	0.140	Soil salinity
NDVI	0.256	0.270	Vegetation productivity

Table S7.Stratified area statistics of habitat-change categories along elevation gradients.

species	period	scenario	change_category	<500 m	500-1000 m	1000-2000 m	>2000 m
Alhagi camelorum	2070s	SSP1-2.6	Stable unsuitable	616.41	2872.96	5521.91	4848.21
Alhagi camelorum	2070s	SSP1-2.6	Expansion	85.85	124.64	174.71	3.17
Alhagi camelorum	2070s	SSP1-2.6	Contraction	0.00	4.83	2.93	0.06
Alhagi camelorum	2070s	SSP1-2.6	Stable suitable	260.08	531.04	613.46	6.52
Alhagi camelorum	2070s	SSP2-4.5	Stable unsuitable	350.17	2592.21	5003.38	4793.03
Alhagi camelorum	2070s	SSP2-4.5	Expansion	352.10	405.39	693.24	58.35
Alhagi camelorum	2070s	SSP2-4.5	Contraction	0.00	0.29	0.29	0.00
Alhagi camelorum	2070s	SSP2-4.5	Stable suitable	260.08	535.59	616.09	6.58
Alhagi camelorum	2070s	SSP3-7.0	Stable unsuitable	347.15	2485.88	4948.53	4780.83
Alhagi camelorum	2070s	SSP3-7.0	Expansion	355.11	511.72	748.08	70.55
Alhagi camelorum	2070s	SSP3-7.0	Contraction	0.00	0.51	0.01	0.00
Alhagi camelorum	2070s	SSP3-7.0	Stable suitable	260.08	535.37	616.38	6.58
Alhagi camelorum	2070s	SSP5-8.5	Stable unsuitable	312.76	2419.39	4877.03	4764.67
Alhagi camelorum	2070s	SSP5-8.5	Expansion	389.51	578.21	819.59	86.71
Alhagi camelorum	2070s	SSP5-8.5	Contraction	0.00	0.00	0.00	0.00
Alhagi camelorum	2070s	SSP5-8.5	Stable suitable	260.08	535.88	616.39	6.58
Alhagi camelorum	2090s	SSP1-2.6	Stable unsuitable	583.45	2849.13	5492.83	4847.59

Alhagi camelorum	2090s	SSP1-2.6	Expansion	118.82	148.48	203.79	3.79
Alhagi camelorum	2090s	SSP1-2.6	Contraction	0.00	4.60	4.90	0.01
Alhagi camelorum	2090s	SSP1-2.6	Stable suitable	260.08	531.28	611.49	6.57
Alhagi camelorum	2090s	SSP2-4.5	Stable unsuitable	273.48	2321.96	4700.52	4755.50
Alhagi camelorum	2090s	SSP2-4.5	Expansion	428.79	675.64	996.09	95.88
Alhagi camelorum	2090s	SSP2-4.5	Contraction	0.00	0.42	0.00	0.00
Alhagi camelorum	2090s	SSP2-4.5	Stable suitable	260.08	535.45	616.39	6.58
Alhagi camelorum	2090s	SSP3-7.0	Stable unsuitable	215.12	2213.86	4642.19	4689.62
Alhagi camelorum	2090s	SSP3-7.0	Expansion	487.15	783.74	1054.42	161.76
Alhagi camelorum	2090s	SSP3-7.0	Contraction	0.00	0.00	0.00	0.00
Alhagi camelorum	2090s	SSP3-7.0	Stable suitable	260.08	535.88	616.39	6.58
Alhagi camelorum	2090s	SSP5-8.5	Stable unsuitable	340.29	2447.15	4756.53	4697.82
Alhagi camelorum	2090s	SSP5-8.5	Expansion	361.98	550.45	940.08	153.56
Alhagi camelorum	2090s	SSP5-8.5	Contraction	0.00	0.00	0.00	0.00
Alhagi camelorum	2090s	SSP5-8.5	Stable suitable	260.08	535.88	616.39	6.58
Glycyrrhiza inflata	2070s	SSP1-2.6	Stable unsuitable	711.26	2529.93	4907.11	4247.30
Glycyrrhiza inflata	2070s	SSP1-2.6	Expansion	97.17	678.33	705.73	538.23
Glycyrrhiza inflata	2070s	SSP1-2.6	Contraction	0.00	0.00	0.01	0.00
Glycyrrhiza inflata	2070s	SSP1-2.6	Stable suitable	153.91	325.22	700.16	72.43
Glycyrrhiza inflata	2070s	SSP2-4.5	Stable unsuitable	463.37	1748.14	3642.40	3779.89
Glycyrrhiza inflata	2070s	SSP2-4.5	Expansion	345.06	1460.12	1970.44	1005.63
Glycyrrhiza inflata	2070s	SSP2-4.5	Contraction	0.00	0.00	0.05	0.00
Glycyrrhiza inflata	2070s	SSP2-4.5	Stable suitable	153.91	325.22	700.11	72.43
Glycyrrhiza inflata	2070s	SSP3-7.0	Stable unsuitable	431.83	1558.04	3327.75	3254.44
Glycyrrhiza inflata	2070s	SSP3-7.0	Expansion	376.60	1650.22	2285.09	1531.09
Glycyrrhiza inflata	2070s	SSP3-7.0	Contraction	0.00	0.00	0.53	0.00

Glycyrrhiza inflata	2070s	SSP3-7.0	Stable suitable	153.91	325.22	699.63	72.43
Glycyrrhiza inflata	2070s	SSP5-8.5	Stable unsuitable	370.22	1576.77	3425.80	3251.06
Glycyrrhiza inflata	2070s	SSP5-8.5	Expansion	438.21	1631.49	2187.04	1534.47
Glycyrrhiza inflata	2070s	SSP5-8.5	Contraction	0.00	0.00	0.41	0.00
Glycyrrhiza inflata	2070s	SSP5-8.5	Stable suitable	153.91	325.22	699.75	72.43
Glycyrrhiza inflata	2090s	SSP1-2.6	Stable unsuitable	694.19	2589.13	4941.41	4173.73
Glycyrrhiza inflata	2090s	SSP1-2.6	Expansion	114.24	619.13	671.43	611.80
Glycyrrhiza inflata	2090s	SSP1-2.6	Contraction	0.00	0.00	0.00	0.00
Glycyrrhiza inflata	2090s	SSP1-2.6	Stable suitable	153.91	325.22	700.16	72.43
Glycyrrhiza inflata	2090s	SSP2-4.5	Stable unsuitable	243.25	1356.46	3060.12	3289.43
Glycyrrhiza inflata	2090s	SSP2-4.5	Expansion	565.18	1851.80	2552.72	1496.09
Glycyrrhiza inflata	2090s	SSP2-4.5	Contraction	0.00	0.00	0.00	0.00
Glycyrrhiza inflata	2090s	SSP2-4.5	Stable suitable	153.91	325.22	700.16	72.43
Glycyrrhiza inflata	2090s	SSP3-7.0	Stable unsuitable	309.03	1316.26	3016.48	3060.07
Glycyrrhiza inflata	2090s	SSP3-7.0	Expansion	499.40	1892.00	2596.36	1725.46
Glycyrrhiza inflata	2090s	SSP3-7.0	Contraction	0.00	0.00	0.26	0.00
Glycyrrhiza inflata	2090s	SSP3-7.0	Stable suitable	153.91	325.22	699.91	72.43
Glycyrrhiza inflata	2090s	SSP5-8.5	Stable unsuitable	453.47	1559.57	3390.49	3293.82
Glycyrrhiza inflata	2090s	SSP5-8.5	Expansion	354.96	1648.70	2222.36	1491.71
Glycyrrhiza inflata	2090s	SSP5-8.5	Contraction	0.00	0.00	0.37	0.00
Glycyrrhiza inflata	2090s	SSP5-8.5	Stable suitable	153.91	325.22	699.79	72.43
Trigonella arcuata	2070s	SSP1-2.6	Stable unsuitable	442.83	2808.83	6112.37	4857.91
Trigonella arcuata	2070s	SSP1-2.6	Expansion	9.06	6.05	1.52	0.00
Trigonella arcuata	2070s	SSP1-2.6	Contraction	51.60	147.60	78.91	0.02
Trigonella arcuata	2070s	SSP1-2.6	Stable suitable	458.85	570.99	120.20	0.03
Trigonella arcuata	2070s	SSP2-4.5	Stable unsuitable	372.42	2709.15	6086.51	4857.83

Trigonella arcuata	2070s	SSP2-4.5	Expansion	79.48	105.74	27.38	0.08
Trigonella arcuata	2070s	SSP2-4.5	Contraction	9.05	46.24	27.96	0.01
Trigonella arcuata	2070s	SSP2-4.5	Stable suitable	501.40	672.35	171.15	0.04
Trigonella arcuata	2070s	SSP3-7.0	Stable unsuitable	395.29	2706.77	6089.53	4857.89
Trigonella arcuata	2070s	SSP3-7.0	Expansion	56.60	108.11	24.36	0.02
Trigonella arcuata	2070s	SSP3-7.0	Contraction	16.37	62.38	39.85	0.01
Trigonella arcuata	2070s	SSP3-7.0	Stable suitable	494.08	656.21	159.26	0.04
Trigonella arcuata	2070s	SSP5-8.5	Stable unsuitable	347.73	2620.82	6056.47	4857.73
Trigonella arcuata	2070s	SSP5-8.5	Expansion	104.17	194.07	57.42	0.18
Trigonella arcuata	2070s	SSP5-8.5	Contraction	0.86	23.20	16.04	0.01
Trigonella arcuata	2070s	SSP5-8.5	Stable suitable	509.59	695.39	183.07	0.04
Trigonella arcuata	2090s	SSP1-2.6	Stable unsuitable	450.01	2814.21	6113.00	4857.90
Trigonella arcuata	2090s	SSP1-2.6	Expansion	1.89	0.68	0.89	0.01
Trigonella arcuata	2090s	SSP1-2.6	Contraction	59.42	187.47	81.93	0.02
Trigonella arcuata	2090s	SSP1-2.6	Stable suitable	451.02	531.12	117.19	0.03
Trigonella arcuata	2090s	SSP2-4.5	Stable unsuitable	422.14	2761.05	6103.20	4857.89
Trigonella arcuata	2090s	SSP2-4.5	Expansion	29.75	53.84	10.69	0.02
Trigonella arcuata	2090s	SSP2-4.5	Contraction	24.23	87.94	65.21	0.01
Trigonella arcuata	2090s	SSP2-4.5	Stable suitable	486.22	630.65	133.91	0.04
Trigonella arcuata	2090s	SSP3-7.0	Stable unsuitable	344.11	2655.26	6072.62	4857.81
Trigonella arcuata	2090s	SSP3-7.0	Expansion	107.78	159.62	41.27	0.10
Trigonella arcuata	2090s	SSP3-7.0	Contraction	2.67	35.35	21.22	0.01
Trigonella arcuata	2090s	SSP3-7.0	Stable suitable	507.78	683.24	177.90	0.04
Trigonella arcuata	2090s	SSP5-8.5	Stable unsuitable	351.06	2638.44	6074.66	4857.81
Trigonella arcuata	2090s	SSP5-8.5	Expansion	100.83	176.44	39.23	0.10
Trigonella arcuata	2090s	SSP5-8.5	Contraction	5.31	36.24	21.38	0.01

Trigonella arcuata	2090s	SSP5-8.5	Stable suitable	505.13	682.35	177.74	0.04

Note: Area values are expressed as ×10² km². Elevation was classified into four gradient classes: <500 m, 500–1000 m, 1000–2000 m, and >2000 m. Stable unsuitable areas were included for completeness.

Table S8.Stratified area statistics of habitat-change categories along soil-salinity gradients.

species	period	scenario	change_category	ECE <2 dS/m	ECE 2-4 dS/m	ECE 4-8 dS/m	ECE >8 dS/m
Alhagi camelorum	2070s	SSP1-2.6	Stable unsuitable	10875.07	2539.93	8.94	435.55
Alhagi camelorum	2070s	SSP1-2.6	Expansion	289.87	48.68	0.00	49.83
Alhagi camelorum	2070s	SSP1-2.6	Contraction	7.49	0.00	0.00	0.34
Alhagi camelorum	2070s	SSP1-2.6	Stable suitable	1038.28	162.28	0.00	210.53
Alhagi camelorum	2070s	SSP2-4.5	Stable unsuitable	10125.50	2293.39	8.94	310.95
Alhagi	2070s	SSP2-4.5	Expansion	1039.43	295.22	0.00	174.42

camelorum							
Alhagi camelorum	2070s	SSP2-4.5	Contraction	0.58	0.00	0.00	0.00
Alhagi camelorum	2070s	SSP2-4.5	Stable suitable	1045.19	162.28	0.00	210.87
Alhagi camelorum	2070s	SSP3-7.0	Stable unsuitable	9952.08	2292.66	8.94	308.72
Alhagi camelorum	2070s	SSP3-7.0	Expansion	1212.86	295.96	0.00	176.66
Alhagi camelorum	2070s	SSP3-7.0	Contraction	0.49	0.00	0.00	0.03
Alhagi camelorum	2070s	SSP3-7.0	Stable suitable	1045.28	162.28	0.00	210.84
Alhagi camelorum	2070s	SSP5-8.5	Stable unsuitable	9802.07	2265.00	8.94	297.84
Alhagi camelorum	2070s	SSP5-8.5	Expansion	1362.86	323.62	0.00	187.54
Alhagi camelorum	2070s	SSP5-8.5	Contraction	0.00	0.00	0.00	0.00
Alhagi camelorum	2070s	SSP5-8.5	Stable suitable	1045.77	162.28	0.00	210.87
Alhagi camelorum	2090s	SSP1-2.6	Stable unsuitable	10805.85	2532.59	8.94	425.62
Alhagi camelorum	2090s	SSP1-2.6	Expansion	359.09	56.02	0.00	59.76
Alhagi	2090s	SSP1-2.6	Contraction	9.17	0.01	0.00	0.33

camelorum							
Alhagi camelorum	2090s	SSP1-2.6	Stable suitable	1036.60	162.27	0.00	210.54
Alhagi camelorum	2090s	SSP2-4.5	Stable unsuitable	9653.23	2139.56	8.94	249.73
Alhagi camelorum	2090s	SSP2-4.5	Expansion	1511.70	449.06	0.00	235.65
Alhagi camelorum	2090s	SSP2-4.5	Contraction	0.38	0.00	0.00	0.04
Alhagi camelorum	2090s	SSP2-4.5	Stable suitable	1045.38	162.28	0.00	210.84
Alhagi camelorum	2090s	SSP3-7.0	Stable unsuitable	9483.96	2030.43	8.94	237.46
Alhagi camelorum	2090s	SSP3-7.0	Expansion	1680.98	558.19	0.00	247.91
Alhagi camelorum	2090s	SSP3-7.0	Contraction	0.00	0.00	0.00	0.00
Alhagi camelorum	2090s	SSP3-7.0	Stable suitable	1045.77	162.28	0.00	210.87
Alhagi camelorum	2090s	SSP5-8.5	Stable unsuitable	9731.37	2226.18	8.94	275.31
Alhagi camelorum	2090s	SSP5-8.5	Expansion	1433.57	362.43	0.00	210.07
Alhagi camelorum	2090s	SSP5-8.5	Contraction	0.00	0.00	0.00	0.00
Alhagi	2090s	SSP5-8.5	Stable suitable	1045.77	162.28	0.00	210.87

camelorum							
Glycyrrhiza inflata	2070s	SSP1-2.6	Stable unsuitable	10032.48	1915.14	8.94	439.05
Glycyrrhiza inflata	2070s	SSP1-2.6	Expansion	1398.83	534.96	0.00	85.67
Glycyrrhiza inflata	2070s	SSP1-2.6	Contraction	0.01	0.00	0.00	0.00
Glycyrrhiza inflata	2070s	SSP1-2.6	Stable suitable	779.38	300.80	0.00	171.53
Glycyrrhiza inflata	2070s	SSP2-4.5	Stable unsuitable	8172.63	1095.19	8.94	357.05
Glycyrrhiza inflata	2070s	SSP2-4.5	Expansion	3258.68	1354.91	0.00	167.67
Glycyrrhiza inflata	2070s	SSP2-4.5	Contraction	0.05	0.00	0.00	0.00
Glycyrrhiza inflata	2070s	SSP2-4.5	Stable suitable	779.34	300.80	0.00	171.53
Glycyrrhiza inflata	2070s	SSP3-7.0	Stable unsuitable	7254.19	959.40	8.94	349.53
Glycyrrhiza inflata	2070s	SSP3-7.0	Expansion	4177.12	1490.69	0.00	175.19
Glycyrrhiza inflata	2070s	SSP3-7.0	Contraction	0.53	0.00	0.00	0.00
Glycyrrhiza inflata	2070s	SSP3-7.0	Stable suitable	778.86	300.80	0.00	171.53
Glycyrrhiza	2070s	SSP5-8.5	Stable unsuitable	7238.84	1032.34	8.94	343.74

inflata							
Glycyrrhiza inflata	2070s	SSP5-8.5	Expansion	4192.47	1417.76	0.00	180.98
Glycyrrhiza inflata	2070s	SSP5-8.5	Contraction	0.41	0.00	0.00	0.00
Glycyrrhiza inflata	2070s	SSP5-8.5	Stable suitable	778.98	300.80	0.00	171.53
Glycyrrhiza inflata	2090s	SSP1-2.6	Stable unsuitable	10033.54	1917.83	8.94	438.16
Glycyrrhiza inflata	2090s	SSP1-2.6	Expansion	1397.77	532.26	0.00	86.56
Glycyrrhiza inflata	2090s	SSP1-2.6	Contraction	0.00	0.00	0.00	0.00
Glycyrrhiza inflata	2090s	SSP1-2.6	Stable suitable	779.39	300.80	0.00	171.53
Glycyrrhiza inflata	2090s	SSP2-4.5	Stable unsuitable	6804.08	809.28	8.94	326.97
Glycyrrhiza inflata	2090s	SSP2-4.5	Expansion	4627.23	1640.81	0.00	197.75
Glycyrrhiza inflata	2090s	SSP2-4.5	Contraction	0.00	0.00	0.00	0.00
Glycyrrhiza inflata	2090s	SSP2-4.5	Stable suitable	779.39	300.80	0.00	171.53
Glycyrrhiza inflata	2090s	SSP3-7.0	Stable unsuitable	6567.65	796.39	8.94	328.86
Glycyrrhiza	2090s	SSP3-7.0	Expansion	4863.66	1653.70	0.00	195.86

inflata							
Glycyrrhiza inflata	2090s	SSP3-7.0	Contraction	0.26	0.00	0.00	0.00
Glycyrrhiza inflata	2090s	SSP3-7.0	Stable suitable	779.14	300.80	0.00	171.53
Glycyrrhiza inflata	2090s	SSP5-8.5	Stable unsuitable	7282.84	1055.32	8.94	350.24
Glycyrrhiza inflata	2090s	SSP5-8.5	Expansion	4148.47	1394.77	0.00	174.48
Glycyrrhiza inflata	2090s	SSP5-8.5	Contraction	0.37	0.00	0.00	0.00
Glycyrrhiza inflata	2090s	SSP5-8.5	Stable suitable	779.02	300.80	0.00	171.53
Trigonella arcuata	2070s	SSP1-2.6	Stable unsuitable	10857.89	2720.87	8.94	634.25
Trigonella arcuata	2070s	SSP1-2.6	Expansion	16.10	0.13	0.00	0.39
Trigonella arcuata	2070s	SSP1-2.6	Contraction	259.62	5.37	0.00	13.14
Trigonella arcuata	2070s	SSP1-2.6	Stable suitable	1077.09	24.52	0.00	48.47
Trigonella arcuata	2070s	SSP2-4.5	Stable unsuitable	10676.72	2712.58	8.94	627.66
Trigonella arcuata	2070s	SSP2-4.5	Expansion	197.27	8.42	0.00	6.98
Trigonella	2070s	SSP2-4.5	Contraction	80.36	1.44	0.00	1.46

arcuata							
Trigonella arcuata	2070s	SSP2-4.5	Stable suitable	1256.35	28.44	0.00	60.15
Trigonella arcuata	2070s	SSP3-7.0	Stable unsuitable	10693.20	2716.29	8.94	631.05
Trigonella arcuata	2070s	SSP3-7.0	Expansion	180.79	4.72	0.00	3.59
Trigonella arcuata	2070s	SSP3-7.0	Contraction	111.42	2.32	0.00	4.87
Trigonella arcuata	2070s	SSP3-7.0	Stable suitable	1225.29	27.56	0.00	56.74
Trigonella arcuata	2070s	SSP5-8.5	Stable unsuitable	10545.70	2704.38	8.94	623.71
Trigonella arcuata	2070s	SSP5-8.5	Expansion	328.29	16.63	0.00	10.93
Trigonella arcuata	2070s	SSP5-8.5	Contraction	38.43	1.16	0.00	0.52
Trigonella arcuata	2070s	SSP5-8.5	Stable suitable	1298.28	28.72	0.00	61.10
Trigonella arcuata	2090s	SSP1-2.6	Stable unsuitable	10871.17	2720.70	8.94	634.31
Trigonella arcuata	2090s	SSP1-2.6	Expansion	2.82	0.31	0.00	0.33
Trigonella arcuata	2090s	SSP1-2.6	Contraction	312.17	4.98	0.00	11.70
Trigonella	2090s	SSP1-2.6	Stable suitable	1024.54	24.91	0.00	49.91

arcuata							
Trigonella arcuata	2090s	SSP2-4.5	Stable unsuitable	10782.80	2719.21	8.94	633.32
Trigonella arcuata	2090s	SSP2-4.5	Expansion	91.20	1.80	0.00	1.31
Trigonella arcuata	2090s	SSP2-4.5	Contraction	165.66	4.25	0.00	7.46
Trigonella arcuata	2090s	SSP2-4.5	Stable suitable	1171.04	25.63	0.00	54.15
Trigonella arcuata	2090s	SSP3-7.0	Stable unsuitable	10589.00	2710.20	8.94	621.67
Trigonella arcuata	2090s	SSP3-7.0	Expansion	284.99	10.81	0.00	12.97
Trigonella arcuata	2090s	SSP3-7.0	Contraction	57.57	1.09	0.00	0.58
Trigonella arcuata	2090s	SSP3-7.0	Stable suitable	1279.14	28.79	0.00	61.04
Trigonella arcuata	2090s	SSP5-8.5	Stable unsuitable	10581.80	2707.79	8.94	623.45
Trigonella arcuata	2090s	SSP5-8.5	Expansion	292.20	13.22	0.00	11.19
Trigonella arcuata	2090s	SSP5-8.5	Contraction	60.69	1.50	0.00	0.75
Trigonella arcuata	2090s	SSP5-8.5	Stable suitable	1276.02	28.38	0.00	60.86

Note: Area values are expressed as ×10^2 km^2. Soil salinity was represented by soil electrical conductivity (ECE) and classified into four gradient classes: ECE <2 dS/m, ECE 2–4 dS/m, ECE 4–8 dS/m, and ECE >8 dS/m. Stable unsuitable areas were included for completeness.

Table S9. Sensitivity analysis of Trigonella arcuata under Xinjiang-wide and restricted-domain background settings.

Modeling domain	Occurrence records	Background points	Optimal RM	Optimal FC	AUC	MaxTSS
Xinjiang-wide domain	29	10000	2.5	L	0.947	0.870
Restricted domain	26	10000	4.0	LQHP	0.813	0.509

The restricted domain was defined as a 100-km buffer around the spatially thinned occurrence records of T. arcuata, clipped to the Xinjiang boundary. AUC and MaxTSS values for the restricted-domain model indicate apparent discrimination based on the available occurrence and background data.

Table S10. NDVI-exclusion sensitivity analysis for evaluating the effect of NDVI temporal mismatch on MaxEnt model performance.

species	Model	Predictors	Occurrence records	Optimal RM	Optimal FC	AUC	MaxTSS
Alhagi camelorum	Full model	16	74	1.0	LQ	0.930	0.778
Alhagi camelorum	NDVI-excluded	15	71	1.0	LQ	0.904	0.688
Glycyrrhiza inflata	Full model	16	114	1.5	LQ	0.910	0.691
Glycyrrhiza inflata	NDVI-excluded	15	107	0.5	LQ	0.887	0.611
Trigonella arcuata	Full model	16	29	2.5	L	0.947	0.870
Trigonella arcuata	NDVI-excluded	15	26	4.0	LQ	0.949	0.850

Note: The full models included NDVI summarized from 2000–2024 and bioclimatic variables representing 1970–2000 climate normals. The NDVI-excluded models were fitted using the same workflow after removing NDVI from the predictor set. This analysis was used to evaluate the influence of NDVI temporal mismatch on model performance. Occurrence records indicate records with complete environmental values after raster extraction.

Table S11. Threshold-sensitivity analysis of projected habitat-change areas for Alhagi camelorum and Glycyrrhiza inflata.

species	period	scenario	threshold_method	threshold_value	Expansion	Contraction	Stable.suitable	Net.change
Alhagi_camelorum	2070s	SSP1-2.6	Equal_sensitivity_specificity	0.1381259	461.9834989	8.814306081	1987.353202	453.1691928
Alhagi_camelorum	2070s	SSP1-2.6	MaxTSS	0.211599294	389.7697746	7.927195608	1415.065683	381.842579
Alhagi_camelorum	2070s	SSP1-2.6	Tenth_percentile_training_presence	0.074009559	704.3062937	8.700643429	2951.57363	695.6056503
Alhagi_camelorum	2070s	SSP2-4.5	Equal_sensitivity_specificity	0.1381259	1847.063562	0.819490812	1995.348017	1846.244071
Alhagi_camelorum	2070s	SSP2-4.5	MaxTSS	0.211599294	1511.592645	0.603220106	1422.389658	1510.989425
Alhagi_camelorum	2070s	SSP2-4.5	Tenth_percentile_training_presence	0.074009559	2436.225047	0.794185531	2959.480088	2435.430862
Alhagi_camelorum	2070s	SSP3-7.0	Equal_sensitivity_specificity	0.1381259	2061.373837	0.266344637	1995.901163	2061.107493
Alhagi_camelorum	2070s	SSP3-7.0	MaxTSS	0.211599294	1688.583536	0.527487282	1422.465391	1688.056049
Alhagi_camelorum	2070s	SSP3-7.0	Tenth_percentile_training_presence	0.074009559	2684.016056	0.333718622	2959.940555	2683.682337
Alhagi_camelorum	2070s	SSP5-8.5	Equal_sensitivity_specificity	0.1381259	2194.214287	0	1996.167508	2194.214287
Alhagi_camelorum	2070s	SSP5-8.5	MaxTSS	0.211599294	1876.285919	0	1422.992879	1876.285919
Alhagi_camelorum	2070s	SSP5-8.5	Tenth_percentile_training_presence	0.074009559	2759.708357	0	2960.274274	2759.708357
Alhagi_camelorum	2090s	SSP1-2.6	Equal_sensitivity_specificity	0.1381259	566.0378081	10.32308923	1985.844418	555.7147189
Alhagi_camelorum	2090s	SSP1-2.6	MaxTSS	0.211599294	476.2309132	9.51353241	1413.479346	466.7173808
Alhagi_camelorum	2090s	SSP1-2.6	Tenth_percentile_training_presence	0.074009559	821.0700691	10.96365592	2949.310618	810.1064132
Alhagi_camelorum	2090s	SSP2-4.5	Equal_sensitivity_specificity	0.1381259	2592.80246	0.105527679	1996.06198	2592.696933
Alhagi_camelorum	2090s	SSP2-4.5	MaxTSS	0.211599294	2200.149728	0.428102509	1422.564776	2199.721626
Alhagi_camelorum	2090s	SSP2-4.5	Tenth_percentile_training_presence	0.074009559	3338.312283	0.067289732	2960.206984	3338.244993
Alhagi_camelorum	2090s	SSP3-7.0	Equal_sensitivity_specificity	0.1381259	2902.958975	0	1996.167508	2902.958975
Alhagi_camelorum	2090s	SSP3-7.0	MaxTSS	0.211599294	2491.344286	0	1422.992879	2491.344286
Alhagi_camelorum	2090s	SSP3-7.0	Tenth_percentile_training_presence	0.074009559	3551.234634	0	2960.274274	3551.234634
Alhagi_camelorum	2090s	SSP5-8.5	Equal_sensitivity_specificity	0.1381259	2427.551024	0	1996.167508	2427.551024

species	period	scenario	threshold_method	threshold_value	Expansion	Contraction	Stable.suitable	Net.change
Alhagi_camelorum	2090s	SSP5-8.5	MaxTSS	0.211599294	2009.919528	0	1422.992879	2009.919528
Alhagi_camelorum	2090s	SSP5-8.5	Tenth_percentile_training_presence	0.074009559	3164.843935	0	2960.274274	3164.843935
Glycyrrhiza_inflata	2070s	SSP1-2.6	Equal_sensitivity_specificity	0.174447974	3339.197437	0	2505.473093	3339.197437
Glycyrrhiza_inflata	2070s	SSP1-2.6	MaxTSS	0.308516189	2025.631546	0.006828395	1255.131061	2025.624718
Glycyrrhiza_inflata	2070s	SSP1-2.6	Tenth_percentile_training_presence	0.095745987	3861.832526	0.020479805	4806.606197	3861.812047
Glycyrrhiza_inflata	2070s	SSP2-4.5	Equal_sensitivity_specificity	0.174447974	6412.423613	0.006846238	2505.466247	6412.416767
Glycyrrhiza_inflata	2070s	SSP2-4.5	MaxTSS	0.308516189	4790.733022	0.05417687	1255.083713	4790.678846
Glycyrrhiza_inflata	2070s	SSP2-4.5	Tenth_percentile_training_presence	0.095745987	6117.396375	0.033981411	4806.592695	6117.362394
Glycyrrhiza_inflata	2070s	SSP3-7.0	Equal_sensitivity_specificity	0.174447974	7395.412293	0.006846238	2505.466247	7395.405447
Glycyrrhiza_inflata	2070s	SSP3-7.0	MaxTSS	0.308516189	5852.172719	0.512377575	1254.625512	5851.660341
Glycyrrhiza_inflata	2070s	SSP3-7.0	Tenth_percentile_training_presence	0.095745987	6967.260276	0.054042021	4806.572635	6967.206234
Glycyrrhiza_inflata	2070s	SSP5-8.5	Equal_sensitivity_specificity	0.174447974	7393.290048	0.006846238	2505.466247	7393.283202
Glycyrrhiza_inflata	2070s	SSP5-8.5	MaxTSS	0.308516189	5801.575463	0.420186071	1254.717703	5801.155277
Glycyrrhiza_inflata	2070s	SSP5-8.5	Tenth_percentile_training_presence	0.095745987	6995.897536	0.040933187	4806.585744	6995.856603
Glycyrrhiza_inflata	2090s	SSP1-2.6	Equal_sensitivity_specificity	0.174447974	3395.407832	0	2505.473093	3395.407832
Glycyrrhiza_inflata	2090s	SSP1-2.6	MaxTSS	0.308516189	2022.577146	0	1255.137889	2022.577146
Glycyrrhiza_inflata	2090s	SSP1-2.6	Tenth_percentile_training_presence	0.095745987	3985.883298	0.037685887	4806.588991	3985.845612
Glycyrrhiza_inflata	2090s	SSP2-4.5	Equal_sensitivity_specificity	0.174447974	7776.177909	0	2505.473093	7776.177909
Glycyrrhiza_inflata	2090s	SSP2-4.5	MaxTSS	0.308516189	6475.078724	0	1255.137889	6475.078724
Glycyrrhiza_inflata	2090s	SSP2-4.5	Tenth_percentile_training_presence	0.095745987	7174.648531	0.006727231	4806.619949	7174.641803
Glycyrrhiza_inflata	2090s	SSP3-7.0	Equal_sensitivity_specificity	0.174447974	8057.527664	0.020557172	2505.452536	8057.507107
Glycyrrhiza_inflata	2090s	SSP3-7.0	MaxTSS	0.308516189	6722.389739	0.235313272	1254.902576	6722.154425
Glycyrrhiza_inflata	2090s	SSP3-7.0	Tenth_percentile_training_presence	0.095745987	7481.12464	0.276926772	4806.34975	7480.847713
Glycyrrhiza_inflata	2090s	SSP5-8.5	Equal_sensitivity_specificity	0.174447974	7412.018672	0.020537224	2505.452556	7411.998135
Glycyrrhiza_inflata	2090s	SSP5-8.5	MaxTSS	0.308516189	5727.795778	0.351793821	1254.786096	5727.443984
Glycyrrhiza_inflata	2090s	SSP5-8.5	Tenth_percentile_training_presence	0.095745987	7041.516575	0.148346452	4806.47833	7041.368229

Note: Area values are expressed as ×10² km². Habitat-change areas were recalculated using three threshold criteria: MaxTSS, the 10th percentile training-presence threshold, and the equal sensitivity-specificity threshold. Expansion represents areas unsuitable under current conditions but suitable under future scenarios; contraction represents areas suitable under current conditions but unsuitable under future scenarios; stable suitable represents areas suitable under both current and future conditions. Net change was calculated as expansion minus contraction.

Table S12. Spatial block cross-validation performance of optimized MaxEnt models for the three dryland Fabaceae species.

species	fold	RM	FC	block_size_km	Training_presences	Validation_presences	Training_background	Validation_background	Threshold_method	Threshold_value	Training_TSS_at_threshold	Validation_AUC	TSS	Precision	Recall	F1_score
Alhagi_camelorum	1	1	LQ	250	53	18	7145	2386	Fold-specific training MaxTSS	0.273283935	0.720321639	0.967937972	0.849725249	0.069958848	0.944444444	0.130268199
Alhagi_camelorum	2	1	LQ	250	53	18	7149	2382	Fold-specific training MaxTSS	0.186864489	0.773350013	0.900387163	0.669185558	0.036945813	0.833333333	0.070754717
Alhagi_camelorum	3	1	LQ	250	53	18	7148	2383	Fold-specific training MaxTSS	0.282160935	0.76943544	0.919359351	0.650883573	0.071038251	0.722222222	0.129353234
Alhagi_camelorum	4	1	LQ	250	54	17	7151	2380	Fold-specific training MaxTSS	0.195454293	0.788623192	0.863890262	0.538235294	0.040740741	0.647058824	0.076655052
Glycyrrhiza_inflata	1	1.5	LQ	250	80	27	7175	2393	Fold-specific training MaxTSS	0.339222699	0.650444251	0.937704106	0.711751869	0.117318436	0.777777778	0.203883495
Glycyrrhiza_inflata	2	1.5	LQ	250	80	27	7177	2391	Fold-specific training MaxTSS	0.191629813	0.672667897	0.704571154	0.223988104	0.027777778	0.37037037	0.051679587
Glycyrrhiza_inflata	3	1.5	LQ	250	80	27	7175	2393	Fold-specific training MaxTSS	0.29348487	0.646402439	0.835306682	0.637259909	0.106741573	0.703703704	0.185365854
Glycyrrhiza_inflata	4	1.5	LQ	250	81	26	7177	2391	Fold-specific training MaxTSS	0.251023639	0.690389567	0.857671396	0.517437184	0.029370629	0.807692308	0.056680162
Trigonella_arcuata	1	2.5	L	250	19	7	7165	2390	Fold-specific training MaxTSS	0.404484116	0.818004187	0.984160191	0.699222953	0.12195122	0.714285714	0.208333333
Trigonella_arcuata	2	2.5	L	250	19	7	7166	2389	Fold-specific training MaxTSS	0.376074759	0.844125035	0.944268373	0.821682712	0.016166282	1	0.031818182
Trigonella_arcuata	3	2.5	L	250	20	6	7167	2388	Fold-specific training MaxTSS	0.459140373	0.852239431	0.928531547	0.781825796	0.0390625	0.833333333	0.074626866
Trigonella_arcuata	4	2.5	L	250	20	6	7167	2388	Fold-specific training MaxTSS	0.432509766	0.937491279	0.800111669	0.273450586	0.005514706	0.5	0.010909091

Note: Spatial block cross-validation was conducted using four folds based on a 250-km spatial grid. The threshold used in each fold was a fold-specific MaxTSS threshold derived from the training data only. Precision, recall, and F1-score were calculated using withheld occurrence records and withheld background points. Because true absence data were unavailable, these metrics should be interpreted as presence-background validation metrics.

Table S13. Background-based confusion matrices from spatial block cross-validation.

species	fold	Threshold_method	Threshold_value	TP	FP	TN	FN
Alhagi_camelorum	1	Fold-specific training MaxTSS	0.273283935	17	226	2160	1
Alhagi_camelorum	2	Fold-specific training MaxTSS	0.186864489	15	391	1991	3
Alhagi_camelorum	3	Fold-specific training MaxTSS	0.282160935	13	170	2213	5
Alhagi_camelorum	4	Fold-specific training MaxTSS	0.195454293	11	259	2121	6
Glycyrrhiza_inflata	1	Fold-specific training MaxTSS	0.339222699	21	158	2235	6
Glycyrrhiza_inflata	2	Fold-specific training MaxTSS	0.191629813	10	350	2041	17
Glycyrrhiza_inflata	3	Fold-specific training MaxTSS	0.29348487	19	159	2234	8
Glycyrrhiza_inflata	4	Fold-specific training MaxTSS	0.251023639	21	694	1697	5
Trigonella_arcuata	1	Fold-specific training MaxTSS	0.404484116	5	36	2354	2
Trigonella_arcuata	2	Fold-specific training MaxTSS	0.376074759	7	426	1963	0
Trigonella_arcuata	3	Fold-specific training MaxTSS	0.459140373	5	123	2265	1

Trigonella_arcuata	4	Fold-specific training MaxTSS	0.432509766	3	541	1847	3

Note: TP indicates withheld occurrence records predicted as suitable; FN indicates withheld occurrence records predicted as unsuitable. FP and TN were calculated using withheld background points rather than confirmed absences and should therefore be interpreted as background-based validation quantities.

Table S14. Sampling-bias assessment and bias-file-equivalent sensitivity analysis for the three focal legume species.
Part A. Sampling-effort null model

Species	Occurrence_records	Observed_mean_sampling_effort	Null_mean_sampling_effort	Null_sd_sampling_effort	P_observed_effort_higher_than_null	Observed_mean_nearest_neighbor_km	Null_mean_nearest_neighbor_km	Null_sd_nearest_neighbor_km	P_observed_more_clustered_than_null
Alhagi_camelorum	73	0.390648674	0.277992743	0.026630903	0.001	74.13357692	59.63469358	6.522084715	0.984
Glycyrrhiza_inflata	113	0.422701157	0.276168019	0.021472161	0.001	53.45692291	44.28171191	4.013356425	0.989
Trigonella_arcuata	28	0.424541218	0.277423621	0.042023756	0.002	810.4752487	115.6659042	19.40839585	1

Part B. Bias-file sensitivity analysis

Species	Model_type	Sampling_effort_correction	Occurrence_records	Background_points	RM	FC	AUC	TSS	MaxTSS_threshold	Current_suitable_area_km2	Current_suitable_area_x100_km2	Change_relative_to_primary_area_percent
Alhagi_camelorum	Primary_random_background	No bias file	71	9592	1	LQ	0.931195597	0.747384851	0.216872884	149945.0658	1499.450658	0
Alhagi_camelorum	Bias_corrected_weighted_background	Sampling-effort weighted background	71	9841	1	LQ	0.80381159	0.501662347	0.628321343	58009.62403	580.0962403	-61.31274895
Glycyrrhiza_inflata	Primary_random_background	No bias file	107	9577	1.5	LQ	0.892622902	0.660530145	0.253700618	213325.1674	2133.251674	0
Glycyrrhiza_inflata	Bias_corrected_weighted_background	Sampling-effort weighted background	107	9839	1.5	LQ	0.743238096	0.379716235	0.501418764	413744.4714	4137.444714	93.95014498
Trigonella_arcuata	Primary_random_background	No bias file	26	9584	2.5	L	0.947677218	0.842132721	0.321797486	223125.7415	2231.257415	0
Trigonella_arcuata	Bias_corrected_weighted_background	Sampling-effort weighted background	26	9843	2.5	L	0.906145719	0.720047046	0.33040563	297817.1703	2978.171703	33.47503896

Note: Sample sizes in the sampling-bias analyses were slightly smaller than the final spatially thinned occurrence datasets because records with incomplete environmental or sampling-effort values after raster extraction were excluded.

Table S15. Bootstrap confidence intervals and pairwise comparisons of niche breadth among the three focal legume species.

Part A. Bootstrap confidence intervals of niche breadth

Species	Species_key	Observed_Levins_B2	Bootstrap_mean_B2	Lower_95_CI	Upper_95_CI
Alhagi camelorum	*Alhagi_camelorum*	0.329413304	0.310695955	0.283849084	0.338574141
Glycyrrhiza inflata	*Glycyrrhiza_inflata*	0.315047262	0.302702312	0.274334958	0.330087506
Trigonella arcuata	*Trigonella_arcuata*	0.22507366	0.20361466	0.162320704	0.241293988

Part B. Pairwise bootstrap comparisons of niche breadth

Species_pair	Observed_difference_B2	Lower_95_CI	Upper_95_CI	Bootstrap_P_value
Alhagi camelorum vs Glycyrrhiza inflata	0.014366042	-0.03078765	0.049198827	0.694694695
Alhagi camelorum vs Trigonella arcuata	0.104339644	0.05895204	0.15573436	0
Glycyrrhiza inflata vs Trigonella arcuata	0.089973602	0.052252966	0.147779325	0

Note: Levins' standardized B2 was calculated from occurrence distributions across environmental-variable bins defined by the background environment. Bootstrap confidence intervals were calculated from 999 resampling replicates. Pairwise differences were considered statistically supported when the 95% bootstrap confidence interval did not overlap zero. These comparisons evaluate species-level niche-breadth differences and should not be interpreted as formal tests of life-form effects.

Table S16. Bootstrap confidence intervals and randomization tests for niche overlap among the three focal legume species.

Part A. Bootstrap confidence intervals of niche overlap

Species_pair	Observed_Schoeners_D	Lower_95_CI_D	Upper_95_CI_D	Observed_Hellingers_I	Lower_95_CI_I	Upper_95_CI_I
Alhagi camelorum vs Glycyrrhiza inflata	0.234	0.075	0.206	0.296	0.106	0.272
Alhagi camelorum vs Trigonella arcuata	0.056	0.000	0.095	0.093	0.000	0.136
Glycyrrhiza inflata vs Trigonella arcuata	0.019	0.000	0.037	0.038	0.000	0.071

Part B. Niche equivalency randomization tests

Species_pair	Metric	Observed	Null_mean	Null_sd	P_lower_than_null	P_higher_than_null	P_two_sided
Alhagi camelorum vs Glycyrrhiza inflata	Schoeners_D	0.233908122	0.186723514	0.029417346	0.94	0.075	0.15
Alhagi camelorum vs Glycyrrhiza inflata	Hellingers_I	0.296040505	0.237822659	0.036035871	0.939	0.066	0.132
Alhagi camelorum vs Trigonella arcuata	Schoeners_D	0.056338028	0.075216061	0.026639605	0.331	0.828	0.662
Alhagi camelorum vs Trigonella arcuata	Hellingers_I	0.093098813	0.11153327	0.037208815	0.339	0.671	0.678
Glycyrrhiza inflata vs Trigonella arcuata	Schoeners_D	0.018691589	0.05444697	0.018474224	0.042	0.989	0.084
Glycyrrhiza inflata vs Trigonella arcuata	Hellingers_I	0.037918525	0.103361228	0.033815874	0.039	0.989	0.078

Note: Schoener's D and Hellinger's I range from 0 to 1, with larger values indicating greater niche overlap. Bootstrap confidence intervals were calculated from 999 resampling replicates. Niche equivalency tests were based on 999 randomizations of species labels while preserving sample sizes. A two-sided P value lower than 0.05 indicates that observed niche overlap differs from randomized niche-equivalency expectations.

Table S17. Bootstrap uncertainty and pairwise comparisons of projected net habitat-suitability change among the three focal legume species.

Part A. Observed full-model projected change

Species	Period	Scenario	Expansion	Contraction	Stable_suitable	Net_change
Alhagi camelorum	2070s	SSP1-2.6	323.53	6.71	1713.41	316.82
Alhagi camelorum	2070s	SSP2-4.5	1010.00	0.01	1720.12	1009.99
Alhagi camelorum	2070s	SSP3-7.0	1140.98	0.97	1719.16	1140.01
Alhagi camelorum	2070s	SSP5-8.5	1241.57	0.00	1720.12	1241.57
Alhagi camelorum	2090s	SSP1-2.6	384.70	6.87	1713.25	377.83
Alhagi camelorum	2090s	SSP2-4.5	1300.04	0.01	1720.12	1300.03
Alhagi camelorum	2090s	SSP3-7.0	1413.11	0.00	1720.12	1413.11
Alhagi camelorum	2090s	SSP5-8.5	1195.36	0.00	1720.12	1195.36
Glycyrrhiza inflata	2070s	SSP1-2.6	1006.84	0.03	957.19	1006.81
Glycyrrhiza inflata	2070s	SSP2-4.5	3012.01	0.13	957.09	3011.88
Glycyrrhiza inflata	2070s	SSP3-7.0	3857.11	0.12	957.09	3856.99
Glycyrrhiza inflata	2070s	SSP5-8.5	3682.61	0.18	957.04	3682.43
Glycyrrhiza inflata	2090s	SSP1-2.6	1017.42	0.01	957.21	1017.41
Glycyrrhiza inflata	2090s	SSP2-4.5	4557.68	0.03	957.19	4557.65
Glycyrrhiza inflata	2090s	SSP3-7.0	5001.76	0.25	956.96	5001.50

Glycyrrhiza inflata	2090s	SSP5-8.5	3836.92	0.31	956.91	3836.61
Trigonella arcuata	2070s	SSP1-2.6	13.26	192.94	2179.07	(179.68)
Trigonella arcuata	2070s	SSP2-4.5	169.99	28.86	2343.16	141.13
Trigonella arcuata	2070s	SSP3-7.0	111.60	81.88	2290.14	29.73
Trigonella arcuata	2070s	SSP5-8.5	224.56	12.85	2359.17	211.72
Trigonella arcuata	2090s	SSP1-2.6	12.74	171.93	2200.08	(159.20)
Trigonella arcuata	2090s	SSP2-4.5	84.00	124.96	2247.06	(40.96)
Trigonella arcuata	2090s	SSP3-7.0	200.50	26.46	2345.56	174.04
Trigonella arcuata	2090s	SSP5-8.5	177.07	25.17	2346.84	151.90

Part B. Bootstrap uncertainty of projected change

Species	Period	Scenario	mean_Expansion	lower_Expansion	upper_Expansion	mean_Contraction	lower_Contraction	upper_Contraction	mean_Stable_suitable	lower_Stable_suitable	upper_Stable_suitable	mean_Net_change	lower_Net_change	upper_Net_change
Alhagi camelorum	2070s	SSP1-2.6	342.32	72.59	738.97	25.40	0.00	92.26	1744.27	884.89	2954.97	316.92	6.80	738.78
Alhagi camelorum	2070s	SSP2-4.5	950.46	262.46	2008.81	5.71	0.00	39.81	1763.97	888.63	2968.19	944.75	241.60	2007.98
Alhagi camelorum	2070s	SSP3-7.0	1062.39	262.42	2256.69	9.58	0.00	51.75	1760.10	888.17	2974.45	1052.81	242.11	2254.75
Alhagi camelorum	2070s	SSP5-8.5	1148.61	355.26	2313.04	0.96	0.00	8.37	1768.71	888.65	2984.23	1147.65	353.95	2313.04
Alhagi camelorum	2090s	SSP1-2.6	392.12	90.05	813.75	22.78	0.02	85.77	1746.89	885.62	2953.22	369.34	35.93	813.41
Alhagi	209	SSP	1198.22	284.43	2536.97	6.14	0.00	46.88	1763.53	888.59	2979.38	1192.08	266.84	2536.69

Species	Period	Scenario	mean_Expansion	lower_Expansion	upper_Expansion	mean_Contraction	lower_Contraction	upper_Contraction	mean_Stable_suitable	lower_Stable_suitable	upper_Stable_suitable	mean_Net_change	lower_Net_change	upper_Net_change
camelorum	0s	2-4.5												
Alhagi camelorum	2090s	SSP3-7.0	1311.38	336.41	2711.77	1.44	0.00	13.97	1768.24	888.64	2985.91	1309.94	330.58	2711.76
Alhagi camelorum	2090s	SSP5-8.5	1110.42	301.61	2285.10	1.47	0.00	13.32	1768.21	888.58	2983.62	1108.96	293.11	2285.06
Glycyrrhiza inflata	2070s	SSP1-2.6	2089.37	521.02	3590.34	1.35	0.00	10.93	1906.53	957.84	3682.46	2088.02	519.33	3589.72
Glycyrrhiza inflata	2070s	SSP2-4.5	4528.67	1671.35	6719.85	1.24	0.02	5.97	1906.64	957.42	3679.25	4527.43	1669.63	6719.73
Glycyrrhiza inflata	2070s	SSP3-7.0	5341.00	1994.54	7954.54	0.42	0.00	3.08	1907.46	958.07	3682.96	5340.57	1994.29	7954.45
Glycyrrhiza inflata	2070s	SSP5-8.5	5168.46	1914.36	7671.96	0.79	0.00	5.13	1907.09	957.99	3682.96	5167.68	1913.75	7671.92
Glycyrrhiza inflata	2090s	SSP1-2.6	2037.50	531.60	3513.71	0.54	0.00	4.25	1907.34	957.97	3682.70	2036.96	530.04	3512.85
Glycyrrhiza inflata	2090s	SSP2-4.5	5813.43	2426.40	8384.65	0.12	0.00	0.83	1907.76	958.20	3682.96	5813.30	2426.15	8384.65
Glycyrrhiza inflata	2090s	SSP3-7.0	6056.34	2477.60	8805.97	0.49	0.00	2.59	1907.40	958.03	3682.96	6055.85	2477.26	8805.94
Glycyrr	209	SSP	5335.89	1919.12	7998.72	1.27	0.00	8.88	1906.61	957.93	3682.96	5334.63	1918.33	7998.66

Species	Period	Scenario	mean_Expansion	lower_Expansion	upper_Expansion	mean_Contraction	lower_Contraction	upper_Contraction	mean_Stable_suitable	lower_Stable_suitable	upper_Stable_suitable	mean_Net_change	lower_Net_change	upper_Net_change
hiza inflata	0s	5-8.5												
Trigonella arcuata	2070s	SSP1-2.6	16.37	0.60	46.80	188.21	75.38	331.62	1826.96	890.32	2517.99	(171.85)	(329.17)	(46.36)
Trigonella arcuata	2070s	SSP2-4.5	173.99	55.74	376.32	42.42	5.46	159.05	1972.75	1073.08	2640.84	131.58	(101.91)	368.80
Trigonella arcuata	2070s	SSP3-7.0	129.14	10.47	403.34	88.08	12.17	229.10	1927.09	1018.54	2595.11	41.06	(208.61)	384.15
Trigonella arcuata	2070s	SSP5-8.5	239.05	70.10	548.04	32.98	3.58	143.06	1982.19	1081.78	2647.25	206.06	(62.99)	538.24
Trigonella arcuata	2090s	SSP1-2.6	11.55	0.31	35.93	194.67	60.22	377.48	1820.50	821.75	2519.50	(183.12)	(376.93)	(38.18)
Trigonella arcuata	2090s	SSP2-4.5	88.87	1.79	354.40	140.85	26.37	354.34	1874.32	930.56	2502.60	(51.98)	(351.43)	320.08
Trigonella arcuata	2090s	SSP3-7.0	234.76	54.83	605.47	42.18	5.85	165.08	1972.99	1078.67	2649.84	192.58	(87.95)	602.24
Trigonella arcuata	2090s	SSP5-8.5	212.91	53.59	473.69	38.43	5.22	149.43	1976.74	1080.35	2661.03	174.48	(100.04)	463.25

Part C. Pairwise comparisons of projected net change

Period	Scenario	Species_pair	Observed_difference_net_change	Mean_difference_net_change	Lower_95_CI	Upper_95_CI	Bootstrap_P_value
2070s	SSP1-2.6	*Alhagi camelorum minus Glycyrrhiza inflata*	(689.99)	(1771.10)	(3451.44)	(291.63)	0.02
2070s	SSP1-2.6	*Alhagi camelorum minus Trigonella arcuata*	496.50	488.76	149.96	946.55	0.00
2070s	SSP1-2.6	*Glycyrrhiza inflata minus Trigonella arcuata*	1186.49	2259.87	720.16	3732.70	0.00
2070s	SSP2-4.5	*Alhagi camelorum minus Glycyrrhiza inflata*	(2001.89)	(3582.68)	(6369.89)	(699.66)	0.02
2070s	SSP2-4.5	*Alhagi camelorum minus Trigonella arcuata*	868.86	813.17	75.29	1873.63	0.02
2070s	SSP2-4.5	*Glycyrrhiza inflata minus Trigonella arcuata*	2870.75	4395.85	1492.44	6541.96	0.00
2070s	SSP3-7.0	*Alhagi camelorum minus Glycyrrhiza inflata*	(2716.98)	(4287.76)	(7478.29)	(1062.77)	0.02
2070s	SSP3-7.0	*Alhagi camelorum minus Trigonella arcuata*	1110.28	1011.75	144.91	2215.23	0.02
2070s	SSP3-7.0	*Glycyrrhiza inflata minus Trigonella arcuata*	3827.26	5299.51	1873.18	7880.71	0.00
2070s	SSP5-8.5	*Alhagi camelorum minus Glycyrrhiza inflata*	(2440.86)	(4020.03)	(7187.41)	(834.05)	0.02
2070s	SSP5-8.5	*Alhagi camelorum minus Trigonella arcuata*	1029.85	941.58	79.53	2121.01	0.04
2070s	SSP5-8.5	*Glycyrrhiza inflata minus Trigonella arcuata*	3470.71	4961.61	1618.77	7398.14	0.00
2090	SSP1-2	*Alhagi camelorum minus*	(639.58)	(1667.62)	(3395.25)	(304.19)	0.02

Period	Scenario	Species_pair	Observed_difference_net_change	Mean_difference_net_change	Lower_95_CI	Upper_95_CI	Bootstrap_P_value
s	.6	*Glycyrrhiza inflata*					
2090s	SSP1-2.6	*Alhagi camelorum minus Trigonella arcuata*	537.03	552.46	168.78	1053.95	0.00
2090s	SSP1-2.6	*Glycyrrhiza inflata minus Trigonella arcuata*	1176.61	2220.08	781.09	3656.47	0.00
2090s	SSP2-4.5	*Alhagi camelorum minus Glycyrrhiza inflata*	(3257.63)	(4621.23)	(7834.21)	(1308.65)	0.00
2090s	SSP2-4.5	*Alhagi camelorum minus Trigonella arcuata*	1340.99	1244.06	270.34	2577.29	0.00
2090s	SSP2-4.5	*Glycyrrhiza inflata minus Trigonella arcuata*	4598.61	5865.29	2444.11	8469.69	0.00
2090s	SSP3-7.0	*Alhagi camelorum minus Glycyrrhiza inflata*	(3588.39)	(4745.91)	(8203.97)	(1234.48)	0.02
2090s	SSP3-7.0	*Alhagi camelorum minus Trigonella arcuata*	1239.07	1117.36	88.75	2532.48	0.04
2090s	SSP3-7.0	*Glycyrrhiza inflata minus Trigonella arcuata*	4827.46	5863.27	2168.03	8712.76	0.00
2090s	SSP5-8.5	*Alhagi camelorum minus Glycyrrhiza inflata*	(2641.25)	(4225.67)	(7549.41)	(869.80)	0.02
2090s	SSP5-8.5	*Alhagi camelorum minus Trigonella arcuata*	1043.46	934.47	67.99	2129.05	0.04
2090s	SSP5-8.5	*Glycyrrhiza inflata minus Trigonella arcuata*	3684.72	5160.14	1639.44	7888.49	0.00

Note: Area values are expressed as $\times 10^2$ km². Net change was calculated as expansion minus contraction. Bootstrap uncertainty was estimated from 100 bootstrap SDM projections in which occurrence records were resampled with replacement and MaxEnt models were refitted using the optimized RM and FC settings. Pairwise tests compare

species-level projected net changes using bootstrap confidence intervals. These tests should not be interpreted as formal tests of life-form effects because each life-form category was represented by only one species.
