# Supplementary material for: Optimized MaxEnt Models Predict Species-Specific Habitat Suitability Shifts for Three Dryland Fabaceae Species in Xinjiang
Source: Plants (Basel). 2026 Jul 23;15(15):2254. doi: 10.3390/plants15152254 (PMC13468077; doi:10.3390/plants15152254)
Supplement: Supplementary file 1 [file plants-15-02254-s001.zip › Supplementary Materials2.pdf]

Supplementary Materials:

Figure S1. Pearson correlation heatmap of environmental variables used in the MaxEnt models

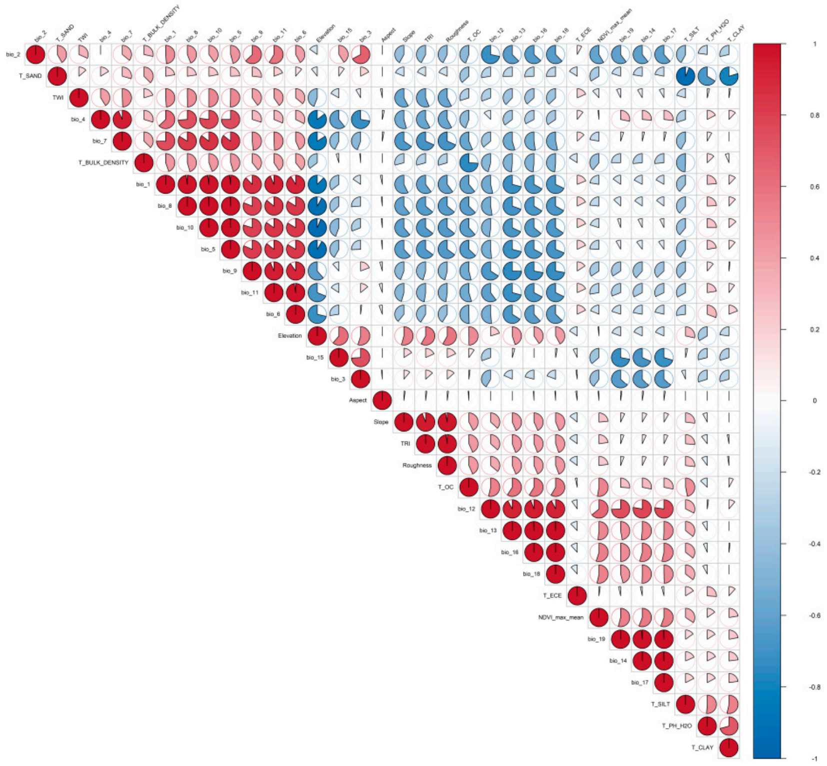

Figure S2. Sampling-effort surface and final thinned occurrence records used for sampling-bias assessment

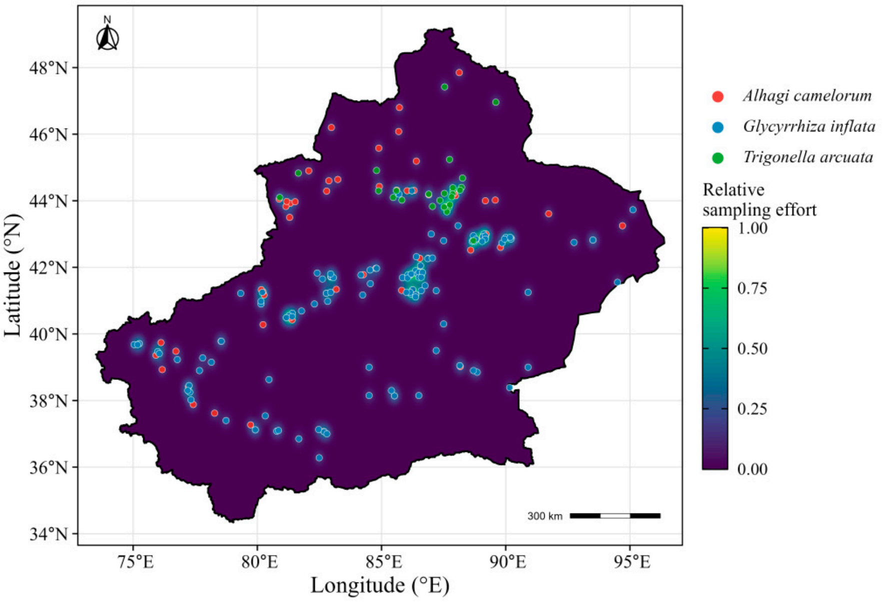

Table S2. Initial VIF values for the 33 candidate environmental predictors

**Environmental factor**	**VIF**
Bio1	551.543041708718
Bio10	1854.32136980198
Bio11	4069.23550884495
Bio12	607.731236794695
Bio13	269.023123322126
Bio14	242.027227367268
Bio15	39.2286258943889
Bio16	626.998935929441
Bio17	611.730748191003
Bio18	472.964002610972
Bio19	285.844563436932
Bio2	1145.89520318981
Bio3	1495.25087882568
Bio4	2909.0745142825
Bio5	9172300666742.35
Bio6	9963715989757.73
Bio7	12941378239570.4
Bio8	48.7751852188592
Bio9	12.5813611881131
Elevation	48.7913135391577
Slope	149.323712867527
Aspect	1.76595247492108
TRI	354.514805763338
Roughness	390.704552834594
TWI	2.77080825202746

PH(H_2O)	6.26554170849843
SOC	4.69543658842554
SAND	Inf
SILT	Inf
CLAY	Inf
BD	2.95514191028751
ECE	3.18350635400481
NDVI	3.38517376138373

Table S3. Stepwise elimination of variables with VIF > 10

Removed_Variable	VIF
SAND	Inf
Bio7	12941378239570.4
Bio11	4069.24
Bio3	1343.44
Bio10	966.73
Bio16	562.53
Bio17	496.41
Bio12	350.19
Roughness	348.84
Bio13	219.12
Bio4	176
Bio14	121.24
Slope	95.04
Bio5	72.08

Bio1	33.24
Bio8	15.72
Bio9	12.5813611881131

Table S4. Final VIF values for the 16 retained environmental predictors

Variable	VIF
Bio15	7.55495785639439
Bio18	7.51693825644006
Bio19	7.87301900829087
Bio2	2.27886158733853
Bio6	6.14776846645972
Elevation	2.29207781958999
Aspect	1.30639668505967
TRI	2.12224771730766
TWI	1.92750774910425
PH(H_2O)	4.68187482481292
SOC	3.35039777663843
SILT	5.41802806153645
CLAY	4.07274262085564
BD	2.06122707888206
ECE	2.11171460734515
NDVI	1.84319064681977
